# Vibronic origin of long-lived coherence in an artificial molecular light harvester

**DOI:** 10.1038/ncomms8755

**Published:** 2015-07-09

**Authors:** James Lim, David Paleček, Felipe Caycedo-Soler, Craig N. Lincoln, Javier Prior, Hans von Berlepsch, Susana F. Huelga, Martin B. Plenio, Donatas Zigmantas, Jürgen Hauer

**Affiliations:** 1 Institut für Theoretische Physik, Universität Ulm, Albert-Einstein Allee 11, 89069 Ulm, Germany; 2 Department of Chemical Physics, Lund University, PO Box 124, SE-22100 Lund, Sweden; 3 Department of Chemical Physics, Charles University in Prague, Ke Karlovu 3, 121 16 Praha 2, Czech Republic; 4 Photonics Institute, Vienna University of Technology, Gusshausstrasse 27, 1040 Vienna, Austria; 5 Departamento de Física Aplicada, Universidad Politécnica de Cartagena, Cartagena 30202, Spain; 6 Forschungszentrum für Elektronenmikroskopie, Institut für Chemie und Biochemie, Freie Universität Berlin, Fabeckstra*β*e 36a, D-14195 Berlin, Germany

## Abstract

Natural and artificial light-harvesting processes have recently gained new interest. Signatures of long-lasting coherence in spectroscopic signals of biological systems have been repeatedly observed, albeit their origin is a matter of ongoing debate, as it is unclear how the loss of coherence due to interaction with the noisy environments in such systems is averted. Here we report experimental and theoretical verification of coherent exciton–vibrational (vibronic) coupling as the origin of long-lasting coherence in an artificial light harvester, a molecular J-aggregate. In this macroscopically aligned tubular system, polarization-controlled 2D spectroscopy delivers an uncongested and specific optical response as an ideal foundation for an in-depth theoretical description. We derive analytical expressions that show under which general conditions vibronic coupling leads to prolonged excited-state coherence.

## Abstract (web summary)

Two-dimensional spectroscopy revealed oscillatory signals in photosynthesis' exciton dynamics, but crowded spectra impede the identification of what sustains the oscillations. Here the authors probe an J-aggregate, whose uncongested response shows that vibronic coupling is responsible for the sustained coherence.

## Graphical abstract

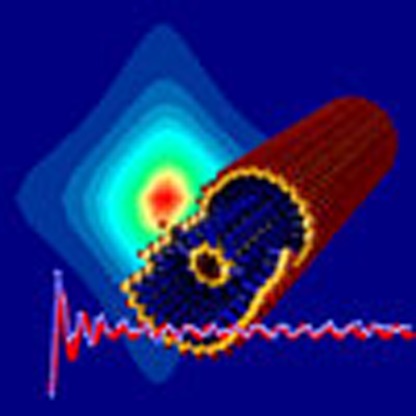

The remarkably high efficiency in photosynthesis, where nine out of ten absorbed photons reach the reaction centre, is a fascinating field of modern research. In such photosynthetic complexes, structure, dynamics and function are inextricably linked. A conserved building block comprises strongly absorbing pigments arranged in close proximity to one another supported by the surrounding protein scaffold^1^,^2^. Typical inter-pigment distances are of order of 10 Å and photon absorption leads to the formation of delocalized excited electronic states (excitons) shared by two or more pigment molecules. Exciton creation, migration and trapping are central to the functionality of a photosynthetic apparatus. The controlled and adjustable arrangement of the pigments tunes the electronic network and the properties of its interaction with the vibrational environment that is associated with either the pigments or the protein. The detailed balance of these properties determines the efficiency of light-harvesting systems^3^,^4^.

Exciton dynamics can be efficiently probed by two-dimensional (2D) electronic spectroscopy^5^. This technique revealed oscillatory signals in the spectral response of a wide variety of photosynthetic aggregates^6^,^7^. Initially ascribed to excitonic beatings, oscillations have been found to persist up to several hundreds of femtoseconds at room temperature^8^,^9^,^10^. This timescale exceeds typical dephasing rates in the condensed phase and becomes comparable to exciton transfer times^1^, thus posing the question of the nature and functional relevance of these coherences^4^. Unfortunately, the complex structure of 2D signals makes the unambiguous identification of the underlying mechanisms that support such long-lived coherences a challenging task and several hypotheses to explain them have been formulated^11^,^12^,^13^,^14^,^15^,^16^,^17^,^18^,^19^,^20^,^21^. The different approaches can be classified into theories including coherent interaction of excitons with intra-pigment vibrations^11^,^12^,^13^,^14^,^15^ and theories focusing on incoherent exciton–protein interaction such as correlated fluctuations^16^,^17^,^18^. It is possible that some of these mechanisms may coexist on certain timescales and that one or another may become dominant depending on the system under consideration.

In this work, we show that the relatively simple excitonic structure of a molecular J-aggregate provides an ideal test case to identify the microscopic mechanism behind long-lived oscillations in electronic 2D signals. The investigated J-aggregate is tubular and aligns along the sample's flow direction when in solution. In addition, the J-aggregate exhibits excitonic bands with roughly orthogonal transition dipole moments. It is this combination of perpendicular excitonic transitions and macroscopic alignment that makes electronic 2D spectroscopy with polarization-controlled excitation pulses an ideal tool to study coherence effects between the excitonic bands. This approach significantly reduces the complexity of retrieved 2D signals, leading to only two peaks with oscillatory components in specific regions of the 2D maps, that is, one on the diagonal and one as a cross-peak for non-rephasing and rephasing signal components, respectively. Employing a vibronic model, we derive analytical expressions that show how system parameters such as electronic decoherence rates and exciton–vibrational resonance determine the amplitude and lifetime of oscillatory signals. Fitting the analytical expressions to measured data, the vibronic model achieves quantitative agreement with experimental observations. Concerning potential functional relevance of the observed oscillations, we show that the long-lived oscillatory signals in our system are dominated by excited-state coherence rather than ground-state coherence.

## Results

### The system

**Figure 1:**
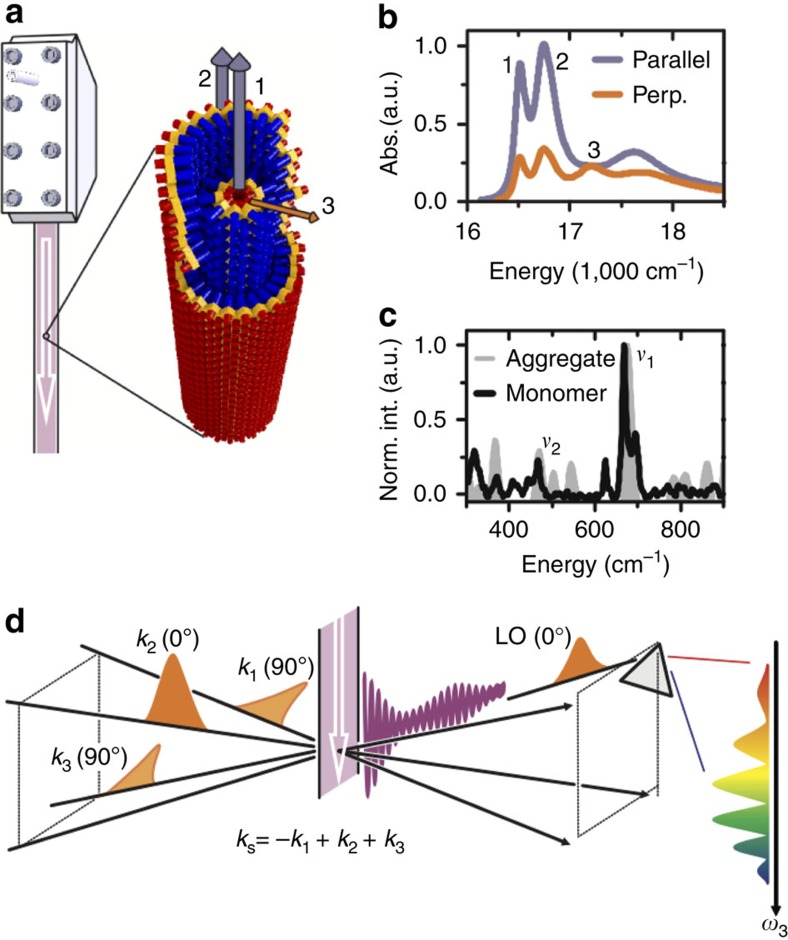
C8O3 and polarization-controlled 2D spectroscopy. (**a**) Wire-guided window-free jet used for sample circulation, along with a schematic of the double-layered structure of the C8O3 aggregate. The aggregates align along the flow direction (white arrow). The transition dipole directions of bands 1–3 are displayed by arrows, which are mainly polarized along the tube axis (bands 1 and 2 shown in blue) or perpendicular to the axis (band 3 shown in orange). (**b**) Absorption spectra in arbitrary units, Abs. (a. u.), with light polarized parallel (blue) and perpendicular (perp.; orange) to the flow direction. (**c**) Non-resonant Raman spectra of the C8O3 monomer (black line) and aggregate (grey area). The vibrational frequencies *ν*_1_ and *ν*_2_ are close to the exciton energy splitting between bands 1 and 3 and bands 2 and 3, respectively. (**d**) Polarization-controlled 2D spectroscopy with three excitation pulses (*k*_1_ to *k*_3_) and a local oscillator (LO) for heterodyne detection of the signal field, depicted as an oscillating line. Polarization orientation (0° or 90°) is given with respect to the longitudinal axis of aligned C8O3.

J-aggregates of cyanine dyes are promising candidates for artificial antenna systems^22^,^23^,^24^,^25^,^26^. They are chemically versatile and self-assemble into various extended supramolecular structures in aqueous solution^27^. Here a system that can be considered a macroscopically aligned synthetic light harvester was studied, namely a molecular J-aggregate of C8O3-monomers whose aggregation behaviour is well known^28^,^29^. As revealed by cryogenic transmission electron microscopy^30^, the aggregate structure is best described as a double-layered nanotube with outer diameter ∼11 nm and lamellar spacing of ∼2.2 nm between the chromophore layers. In addition, superhelical bundles of these tubes can also form though the addition of polyvinyl alcohol inhibits this process and thereby avoids single-layered tube formation^24^ and maintains a stable solution over several weeks^31^. A drawing of the J-aggregate under investigation, from here on referred to as C8O3, is shown in Fig. 1a. The bilayer configuration of C8O3 allows the effect of different decoherence rates to be studied as the outer solvent-exposed layer shows faster decoherence than the inner protected layer.

The structural properties of the aggregate are remarkable: the 11-nm outer diameter is contrasted by a length of several micrometres. Circulating solvated C8O3 with a wire-guided jet (Fig. 1a) leads to a macroscopic orientation of the tubes because the longitudinal axis preferentially aligns along the flow direction. This creates anisotropy for linearly polarized light, as shown in Fig. 1b. Linear dichroism measurements^31^ and redox-chemistry studies^32^ assign bands 1 and 2 to longitudinal transitions localized on the inner and outer cylinders, respectively (Fig. 1a). Transitions to band 3 are preferentially polarized perpendicular to the long axis of C8O3 and are shared by both layers. A detailed description of sample-preparation methods and band assignments is given in the Supplementary Notes 1 and 2.

Fitting the well-defined absorption peaks of C8O3 with Lorentzian functions (see Supplementary Note 2) reveals an exciton energy difference between bands 1 and 3 of ΔΩ_31_≈690 cm^−1^ and ΔΩ_32_≈460 cm^−1^ for bands 2 and 3. Both exciton energy splittings are close to vibrational frequencies *ν*_1_≈668 cm^−1^ and *ν*_2_≈470 cm^−1^ observed in non-resonant Raman spectra^33^ (Fig. 1c). These vibrational frequencies are measured in both the monomer and aggregate Raman spectra, that is, they are not aggregation-induced Raman bands. Strongly enhanced modes at similar energies were observed in resonant Raman spectra of a related cyanine dye, and can be assigned to out-of-plane vibrations^34^. Such out-of-plane vibrations were shown to couple strongly to excitons^35^. The quasi-resonance between the vibrational frequencies *ν*_1_ and *ν*_2_ and exciton energy splittings ΔΩ_31_ and ΔΩ_32_ provides us with an interesting scenario of possible coherent interaction between bands (excitons) and vibrations^11^,^13^,^14^,^21^,^36^. Such exciton–vibrational coupling induces vibronic^12^ and vibrational coherences^15^, which can both lead to long-lived beating signals in 2D spectra. Here we emphasize that coherence in the electronic excited-state manifold is referred to as vibronic and in the ground-state manifold as vibrational. Identifying the dominant contribution is of fundamental importance because only vibronic coherence, which manifests in excited-state dynamics, can enhance exciton transport and thus support light-harvesting function^37^,^38^,^39^.

### Experimental results

The absorption spectrum of a light-harvesting system may be heavily congested because of overlapping excitonic bands and the resulting 2D signal would exhibit significant overlap between diagonal and cross-peaks, thereby impeding further analysis. It has been suggested to employ laser pulses of different relative polarization to selectively address relevant excitation pathways to obtain a clearer 2D signal^40^. However, the advantage of polarization-controlled 2D spectroscopy has been limited by the isotropic nature of the investigated samples (an ensemble). In the experiment presented here, these problems are circumvented by the measurement of the macroscopically aligned C8O3. The transition dipole moments of bands 1 and 2 are preferentially parallel to the longitudinal axis while band 3 is orthogonal, thus allowing for optimal polarization selectivity. This combination reduces the obtained 2D maps to only two relevant peaks with negligible overlap and an up to 30 times stronger signal intensity as compared with the isotropic case^41^.

**Figure 2:**
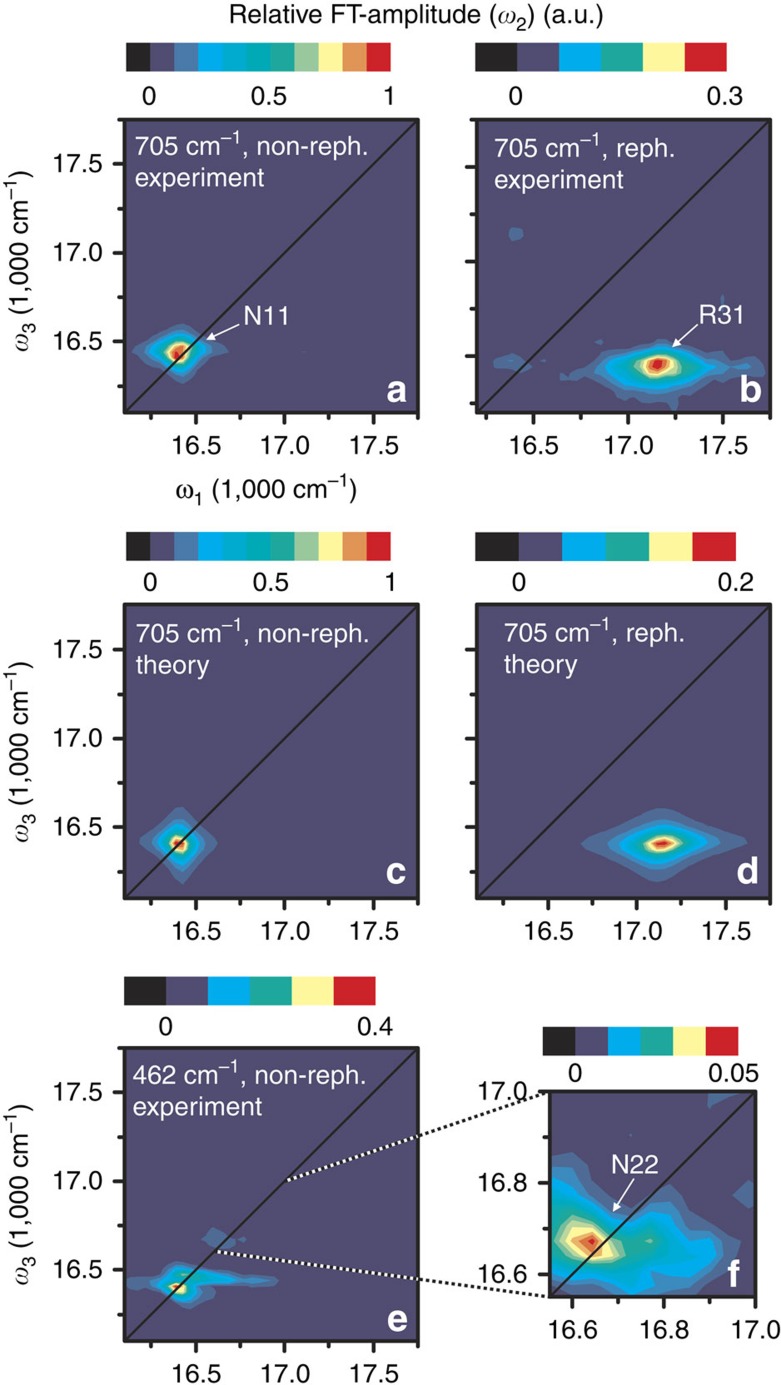
Experimental and theoretical 2D spectra. (**a**,**b**) The Fourier-transform (FT) amplitude maps of non-rephasing (non-reph.) and rephasing (reph.) spectra at *ω*_2_=705±20 cm^−1^, which reveal the presence of a non-rephasing diagonal peak N11 and a rephasing cross-peak R31. These peaks stem from the coherent interaction of bands 1 and 3 with the quasi-resonant vibrational mode with frequency *ν*_1_≈668 cm^−1^. The amplitude of N11 is about three times larger than R31. The lineshape of N11 is symmetric along both *ω*_1_ and *ω*_3_ axes, while that of R31 is elongated along *ω*_1_ axis. (**c**,**d**) The simulated spectra at *ω*_2_=705 cm^−1^ with N11 and R31. (**e**) The FT amplitude map at *ω*_2_=462±20 cm^−1^ reveals coherent interaction of bands 2 and 3 with the quasi-resonant vibrational mode with frequency *ν*_2_≈470 cm^−1^. However, as depicted in (**f**) the associated non-rephasing peak N22 at *ω*_1,3_≈16,670 cm^−1^ is weak and only amounts to 5% of N11 at *ω*_2_=705±20 cm^−1^ (see **a**). The diagonal peak at *ω*_1,3_≈16,400 cm^−1^ in **e** stems from N11, with a peak centred at *ω*_2_=705±20 cm^−1^, but broad enough to appear at *ω*_2_=462±20 cm^−1^. All measurements were carried out at room temperature.

The ideal pulse sequence to isolate beating signals between states with orthogonal transition dipole moments, that is, bands 1 and 3 in the present case, is depicted in Fig. 1d, where the phase-matched direction for measuring rephasing spectra is displayed: non-rephasing spectra can be measured along the same phase-matched signal direction by changing the order of the first two pulses (see Methods). After subtraction of the non-oscillatory background, we performed a Fourier transformation along waiting time *t*_2_ for all points on the 2D (*ω*_1_, *ω*_3_) map. The resulting *ω*_2_ plots allow the lineshape of beating signal with frequency *ω*_2_ to be visualized as a function of position in (*ω*_1_, *ω*_3_) space. The slice at the exciton energy splitting between bands 1 and 3 (*ω*_2_=705±20 cm^−1^ with the experimental resolution of ±20 cm^−1^) reveals a non-rephasing diagonal peak N11 and a rephasing cross-peak R31 as shown in Fig. 2a,b, respectively. N11 is centred at (*ω*_1_, *ω*_3_)=(Ω_1_, Ω_1_) with exciton energy Ω_1_≈16,405 cm^−1^ of band 1 and a symmetric linewidth 2Γ_*g*1_≈130 cm^−1^ along both *ω*_1_ and *ω*_3_ axes (Fig. 2a). The centre of R31 is located at (*ω*_1_, *ω*_3_)=(Ω_3_, Ω_1_) with exciton energy Ω_3_≈17,125 cm^−1^ of band 3 and asymmetric linewidths 2Γ_*g*3_≈300 cm^−1^ and 2Γ_*g*1_≈130 cm^−1^ along *ω*_1_ and *ω*_3_ axes, respectively (Fig. 2b). In peak amplitude, R31 is ∼30% of N11. Turning to the *ω*_2_ slice corresponding to the energy splitting between bands 2 and 3 (*ω*_2_=462±20 cm^−1^), Fig. 2e,f reveal a diagonal non-rephasing peak N22, which is centred at (*ω*_1_, *ω*_3_)=(Ω_2_, Ω_2_) with the exciton energy Ω_2_≈16,672 cm^−1^ of band 2 and a symmetric linewidth 2Γ_*g*2_≈225 cm^−1^ along *ω*_1_ and *ω*_3_ axes. The amplitude of N22 is only 5% of N11.

### Theoretical model

**Figure 3:**
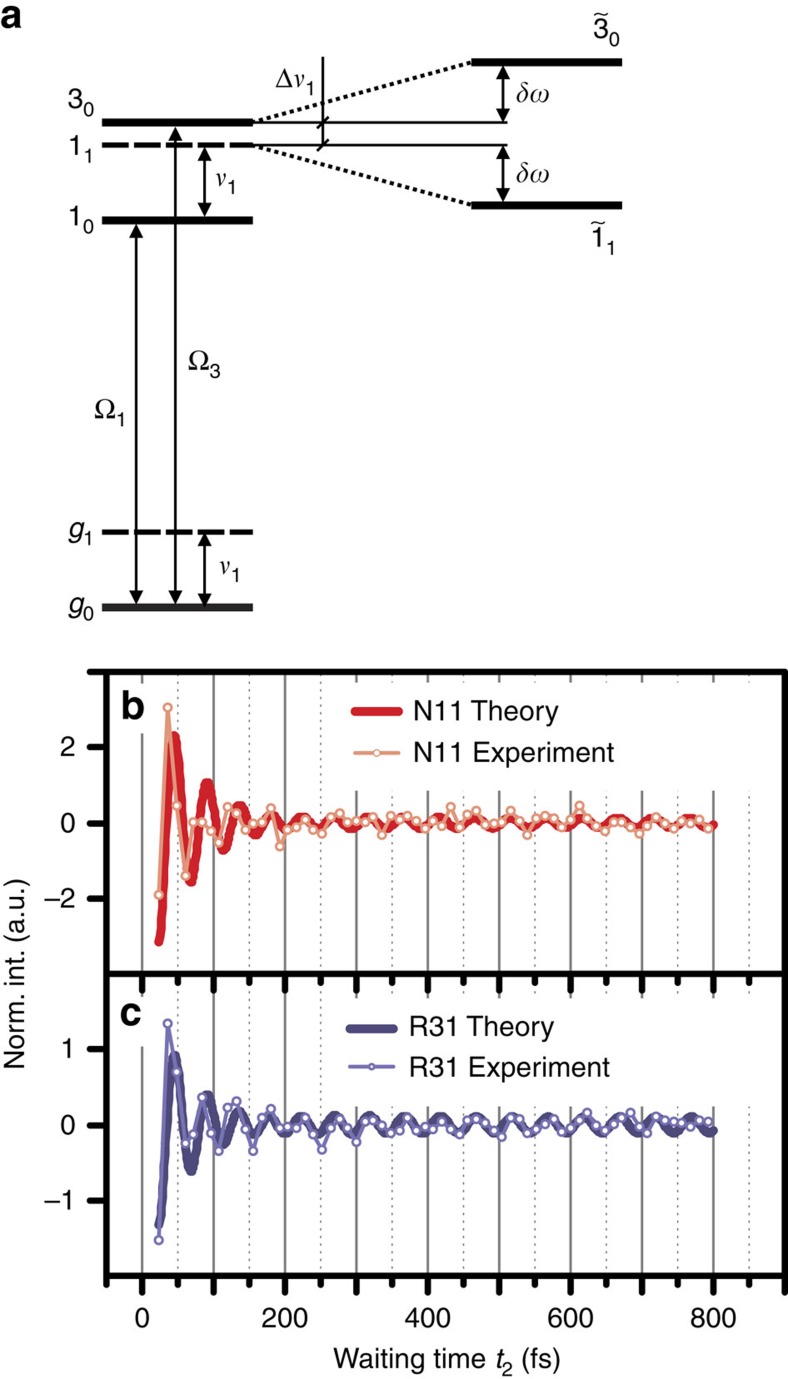
Vibronic model. (**a**) We consider a vibronic model for bands 1 and 3 coupled to a vibrational mode with frequency *ν*_1_≈668 cm^−1^ (see Supplementary Note 2). The vibronic states |*k*_0_〉 and |*k*_1_〉 denote the vibrational ground and first excited states of an electronic state |*k*〉, respectively, with the single index states |*g*〉, |1〉 and |3〉 denoting the electronic ground state and bands 1 and 3, respectively. The exciton energy splitting ΔΩ_31_=Ω_3_−Ω_1_ between bands 1 and 3 is quasi-resonant with the vibrational frequency *ν*_1_, where the detuning is denoted by Δ*ν*_1_=ΔΩ_31_−*ν*_1_. The exciton–vibrational coupling between uncoupled states |3_0_〉 and |1_1_〉 leads to vibronic eigenstates 
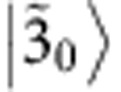
 and 
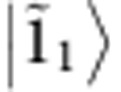
, each of which is a superposition of |3_0_〉 and |1_1_〉, leading to an energy-level shifting by *δω*. (**b**) The time trace of N11 in normalized intensity (Norm. Int.) against waiting time *t*_2_, where the experimental results are shown as light red circles and the theoretical simulation is shown as a full red line. (**c**) The time trace of R31 where the experimental results are shown as light blue circles and the simulated data are depicted as a full blue line. The root mean squared deviation between the experimental results and theoretical simulation in **b** and **c** is 0.92 and 0.59, respectively.

To describe the long-lived oscillations in N11 and R31, a vibronic model is employed that describes the coupling of bands 1 and 3 to a quasi-resonant vibrational mode with frequency *ν*_1_. Consider a system with electronic ground state |*g*_*k*_〉 and excited states for bands 1 and 3, denoted by |1_*k*_〉 and |3_*k*_〉, respectively, where *k*=0 and 1 denote the vibrational ground and excited states, respectively (Fig. 3a). The vibronic coupling between the quasi-resonant states |3_0_〉 and |1_1_〉 leads to unnormalized vibronic eigenstates 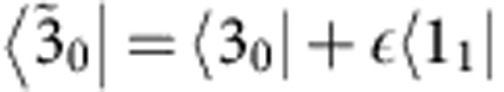 and 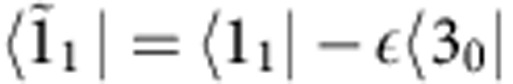. Here 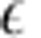 represents the degree of vibronic mixing defined by


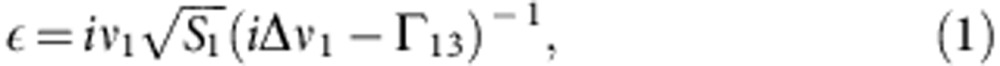


where Δ*ν*_1_=(Ω_3_−Ω_1_)−*ν*_1_ denotes the detuning between |3_0_〉 and |1_1_〉, that is, between the exciton energy splitting and vibrational frequency, and *S*_1_ denotes the Huang–Rhys factor of the vibrational mode, which in turn quantifies the strength of the vibronic coupling (see Supplementary Note 2 for details of the derivation). The electronic decoherence rate Γ_*gk*_ describes the exponential decay rate of the coherence between electronic ground state and band *k*, while Γ_13_ represents the overall exponential decay rate of the inter-exciton coherence between bands 1 and 3. In our model, we do not consider inhomogeneous broadening, which is justified by the observation that the experimentally measured absorption spectrum is well matched to a sum of Lorentzian functions with the linewidths 2Γ_*gk*_ (see Supplementary Note 2). This is valid when homogeneous broadening dominates the linewidths and the Huang–Rhys factors are sufficiently small, as is the case here. In addition, the lineshape of N11 (Fig. 2a) is not elongated along the diagonal *ω*_1_=*ω*_3_, implying our 2D signal is dominated by homogeneous broadening. The same conclusion is reached from analysing 2D correlation spectra^33^.

In nonlinear spectroscopy, the molecular response to laser excitation is described by response functions^42^. According to the vibronic model described above, the response function for the oscillatory signals in N11 reads


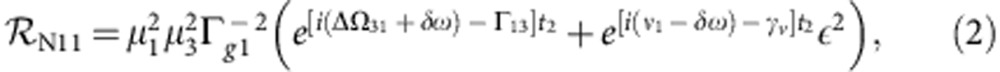


with *μ*_1_ and *μ*_3_ denoting the transition dipole moment of bands 1 and 3, respectively. The prefactor Γ_*g*1_^−2^ stems from the lineshape of N11, *γ*_*v*_ denotes the dissipation rate of the vibrations and *δω* stands for the frequency shift of the vibronic eigenstates 
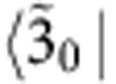
 and 
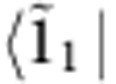
 relative to the uncoupled states 〈3_0_| and 〈1_1_| due to the vibronic coupling (see Fig. 3a and Supplementary Note 2 for further details). The coupling was found to be sufficiently strong to induce non-negligible vibronic mixing 
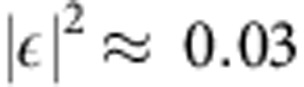
, which leads to a long-lived beating signal in N11 up to *t*_2_≈800 fs, as shown in Fig. 3b. These results imply that the initial excitonic part of 
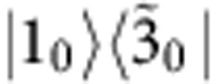
 decays rapidly with 1/*e* decay time of Γ_13_^−1^≈66 fs, while the vibronic coherence 
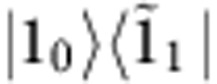
 explains a long-lived oscillatory signal in N11: here 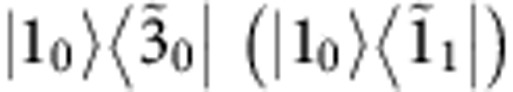 represents coherence between two vibronic states |1_0_〉 and 
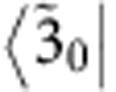
 (|1_0_〉 and 
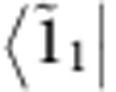
), respectively.

The response function for the oscillatory contributions to R31 is given by


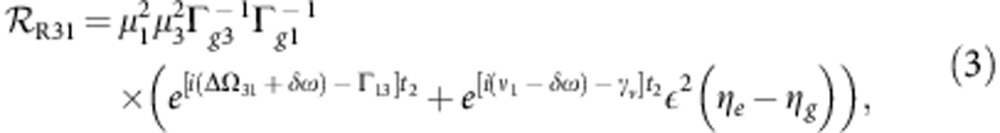


where 
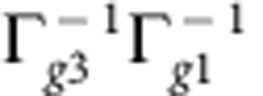
 derives from the asymmetric lineshape of R31 (see Fig. 2b,d). Here *η*_*e*_ and *η*_*g*_ represent the contribution of excited-state vibronic coherence 
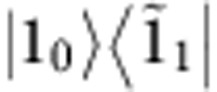
 and ground-state vibrational coherence |*g*_0_〉〈*g*_1_|, respectively, to the long-lived beating signal in R31 (see Supplementary Note 2). The vibrational coherence in the electronic ground-state manifold does not play a role in exciton transfer dynamics, but nonetheless modulates the 2D spectra. A fit of model parameters to experimental results (Fig. 3c) shows that |*η*_*e*_|≈2.5|*η*_*g*_|. This means the long-lived beating signal in R31 is dominated by the excited-state coherence 
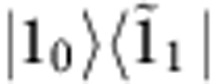
. The short-lived beating signal in R31 is induced by 
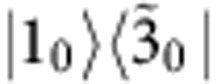
, as is the case for N11. We note that the signal at N11, with approximately three times the amplitude of R31, is exclusively determined by excited-state contributions. Details of this vibronic model and the corresponding Feynman diagrams for the spectral components N11 and R31 are discussed in the Supplementary Note 2.

These results demonstrate how an excitonic system within a noisy environment can exhibit long-lasting coherent features: the observed long-lived oscillations are the result of coherent interaction of excitonic bands with an underdamped, quasi-resonant vibration. This vibronic mechanism requires the vibrational dissipation rate *γ*_*v*_ to be much slower than the electronic decoherence rate Γ_13_, which is the case for C8O3, where 
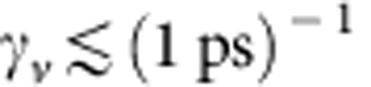
 and Γ_13_≈(66 fs)^−1^. The difference in electronic and vibrational decoherence rates can be rationalized from the fact that excitons and vibrations are related to the motion of electrons and nuclei, respectively. The lower mass of electrons as compared with nuclei makes excitons more mobile and therefore more sensitive to environmental fluctuations, such as local electric fields, than vibrations. We note that the vibronic mixing leading to long-lived beating signals in 2D electronic spectra is described by a vibronic coupling that induces coherent energy exchange between excitons and quasi-resonant vibrations (see Supplementary Note 2 for further details):


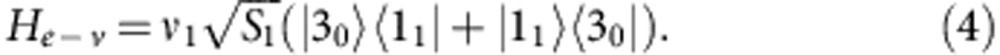


This implies that the vibronic coupling not only induces long-lasting electronic excited-state coherences but also can mediate population transfer between excitonic bands. In a combination with thermal relaxation of exciton populations, the vibronic coupling may further enhance exciton population transfer and as a result could, in principle, have functional relevance in exciton transport^14^,^38^,^43^,^44^,^45^.

Interestingly, the different decoherence rates Γ_*g*3_≈2Γ_*g*1_ of bands 1 and 3 lead to different amplitudes of the short-lived beating signals in N11 and R31 (Fig. 3b,c), which are determined by the prefactors 
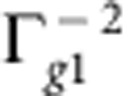
 and 
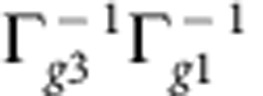
, respectively. The lower decoherence rate of band 1 can be explained by band 1 being localized on the inner layer, while band 3 is delocalized over both the inner and outer layers^46^. As shown by the response functions for N11 and R31, the overall strength of the beating signals is proportional to the inverse of the electronic decoherence rates. It is therefore expected that the beating signal amplitude would diminish with an increase of the decoherence rate. This is the case for N22, where the physical situation in terms of exciton–vibrational resonance (ΔΩ_32_≈*ν*_2_≈470 cm^−1^) is equivalent to N11 (ΔΩ_31_≈*ν*_1_≈668 cm^−1^). The crucial difference is that band 2 has a higher decoherence rate than band 1, as band 2 is localized on the outer layer exposed to solvent^46^. This explains the broader linewidth of band 2 in absorption and 2D spectra. Using an estimated value of Γ_*g*2_≈(47 fs)^−1^, the presented theory predicts the strength of N22 to be 5% of N11 (see Supplementary Note 2), which is in line with the experimental observations (Fig. 2f). These results indicate that the experimentally observed long-lived beating signals, induced by vibronic mixing, require adequately low electronic decoherence rates, highlighting that resonance between exciton energy splitting and vibrational frequency alone is not sufficient^47^.

The presented vibronic model achieves quantitative agreement with the experimental observations. Crucially, the constraints imposed by the observed asymmetric decoherence rates Γ_*g*3_≈2Γ_*g*1_ and fast relaxation of exciton population in C8O3 on sub-picosecond timescales^33^ rule out incoherent models, where long-lived oscillations are sustained by Markovian correlated fluctuations (see Supplementary Note 3 for a detailed analysis). This further supports our conclusion that the observed experimental data provide evidence for vibronic mixing being the mechanism at play in our system.

We note that our results do not imply that correlated fluctuations can be universally ruled out, as this mechanism could be in place in certain pigment–protein complexes. The notion of correlated fluctuations has been developed for photosynthetic complexes where pigments are embedded in a protein scaffold. The protein has been considered as the potential source of correlated fluctuations in natural light harvesters^16^,^17^. For C8O3, a structural frame such as a protein scaffold is absent and therefore correlated fluctuations are unlikely to induce long-lived oscillatory 2D signals, which is in line with our observations.

## Discussion

We have verified, theoretically and experimentally, that coherent vibronic coupling in the electronic excited-state manifold is responsible for the long-lived beating signals observed in 2D spectra of an artificial light harvester. The relatively simple electronic and vibrational structure of the investigated molecular aggregate along with its macroscopic alignment allowed us to rule out the presence of correlated fluctuations. The specific geometry of our system allowed us to gain further insights by illustrating the conditions under which intra-pigment vibrations can prolong electronic coherent effects. The moderately low decoherence rate of band 1, localized on the inner layer and protected from solvent, is the basis for exciton–vibrational coupling as the source of long-lived beating signals. The outer band 2, even though resonantly coupled to a vibration, exhibits a higher decoherence rate and therefore fails to produce observable oscillations. We conclude that the mere resonance between excitons and vibrations does not suffice to explain long-lived beating signals. An adequately low electronic decoherence rate, determined by the interaction between system and bath, is an equally important prerequisite.

The influence of vibronic coupling on energy transport in molecular aggregates has been extensively studied in the past, as recently reviewed^44^. The vibronic coupling has recently gained new interest (see ref. 48 for a recent tutorial overview), as it was suggested as a feasible mechanism to explain long-lived oscillations in the 2D spectra of several natural light-harvesting complexes and a photosynthetic reaction centre^9^,^10^. The requirement of exciton–vibrational resonance is readily satisfied in such systems, given their numerous excitonic bands and rich vibrational structures. Incoherent models based on correlated fluctuations were not ruled out though. Our work provides a quantum mechanical foundation for enhanced energy transfer based on vibronic coupling. As recently demonstrated, this mechanism is not limited to natural light harvesting, vibronic coupling is also of key importance in photovoltaic devices^49^.

## Methods

### Polarization-controlled 2D electronic spectroscopy

In 2D electronic spectroscopy, three ultrashort laser pulses generate an optical response of a molecular ensemble, which is spectrally resolved along both absorption (*ω*_1_) and detection (*ω*_3_) frequencies within the laser pulse spectrum. The absorption frequency *ω*_1_ is obtained by precise scanning of the time delay between the first two pulses and subsequent Fourier transformation (*t*_1_→*ω*_1_). In detection, the signal is spectrally dispersed, leading directly to the detection frequency *ω*_3_. Varying time delay *t*_2_ between pulses 2 and 3 provides information about evolution of the system on a femtosecond timescale^50^,^51^,^52^. To retrieve the purely absorptive part, the signal induced by pulses 1–3 is detected in a heterodyned fashion by interfering it with a phase-stable local oscillator pulse. Polarization control is achieved by the combination of *λ*/4 wave plates and wire grid polarizers for each of the laser beams to select the desired polarization with high accuracy. Polarization-resolved 2D experiments change the relative contributions of distinct pathways depending on the polarization of the laser pulses, orientation of the transition dipole moments and isotropy of the sample^40^. Rephasing spectra were acquired with a polarization sequence of (90°, 0°, 90°, 0°) for pulses (1, 2, 3, local oscillator), in contrast to non-rephasing spectra, where the time ordering of the first two pulses is reversed, leading to a polarization sequence of (0°, 90°, 90°, 0°). The polarization scheme used for rephasing spectra (Fig. 1d) shows 0° was defined to be parallel to the sample flow direction, depicted as a white arrow in Fig. 1a. For a macroscopically aligned sample, this particular polarization sequence selects pathways stemming from interband coherences and vibronic mixing^12^,^15^, discussed throughout the paper, while pathways with all-parallel transition dipole moments such as ground-state bleach, stimulated emission, excited-state absorption and also vibrational wave packet excitation are suppressed. For the details regarding the experimental methods, see Supplementary Note 1. To subtract the non-oscillatory signals from 2D spectra, we employed a decay-associated spectra analysis^33^, where the population decays were fitted by a sum of three 2D spectra with individual decay constants. The *ω*_2_ maps in Fig. 2 were obtained using Fourier transformation (*t*_2_→*ω*_2_) with zero-padding up to 2^7^ data points. All measurements were carried out at room temperature.

## Additional information

**How to cite this article**: Lim, J. *et al.* Vibronic origin of long-lived coherence in an artificial molecular light harvester. *Nat. Commun.* 6:7755 doi: 10.1038/ncomms8755 (2015).

## Supplementary Material

Supplementary Information Supplementary Figures 1-7, Supplementary Notes 1-3 and Supplementary References
