# Supplementary material for: Vibronic origin of long-lived coherence in an artificial molecular light harvester
Source: Nat Commun. 2015 Jul 9;6:7755. doi: 10.1038/ncomms8755 (PMC4510969; doi:10.1038/ncomms8755)
Supplement: Supplementary Information — Supplementary Figures 1-7, Supplementary Notes 1-3 and Supplementary References [file ncomms8755-s1.pdf]

Supplementary Figures:

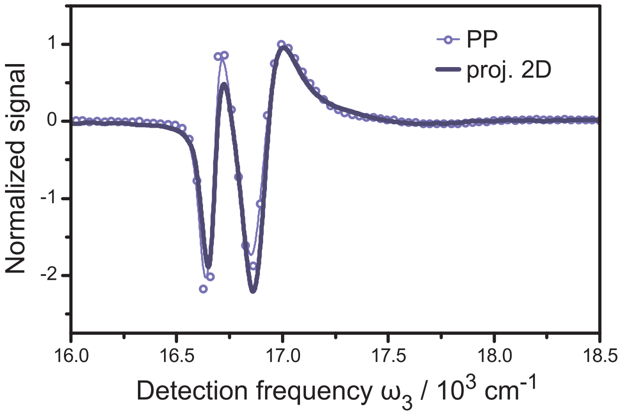

Supplementary Figure 1. **Pump-probe and projected 2D signal**. Projection of phased polarization-controlled 2D spectra (blue) to all-parallel pump-probe (light blue) at t_2 = 180 fs.

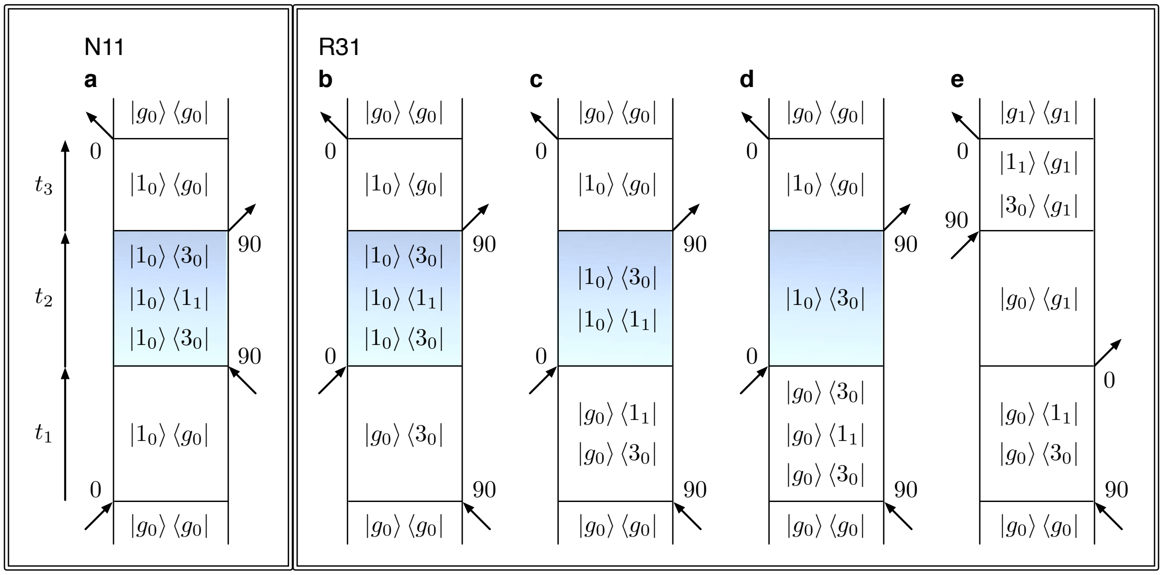

Supplementary Figure 2. **Feynman diagrams contributing to the beating signals in N11 and R31 represented in uncoupled state basis**. **a**, The stimulated emission diagram contributing to the beating signals in N11. Here time runs upwards and the electronic transitions induced by light are denoted by arrows: 0 and 90 denote the polarization of light, parallel and normal to the longitudinal axis of C8O3, respectively (*cf.* Figure 1 in the main text). The time interval between pulses is called coherence time t_1, waiting time t_2, and rephasing time t_3 for the first and second, the second and third, and the third excitation pulse and the emerging signal, respectively. The Fourier transform along t_1 and t_3 leads to the absorption and detection frequencies denoted by ω_1 and ω_3, respectively. **b-e**, The stimulated emission and ground state bleaching diagrams contributing to the beating signals in R31. In **a-d**, grey shaded waiting time periods during t_2 highlight vibronic coherences in the electronic excited states. In **e**, on the other hand, the vibronic system is in the electronic ground state during t_2.

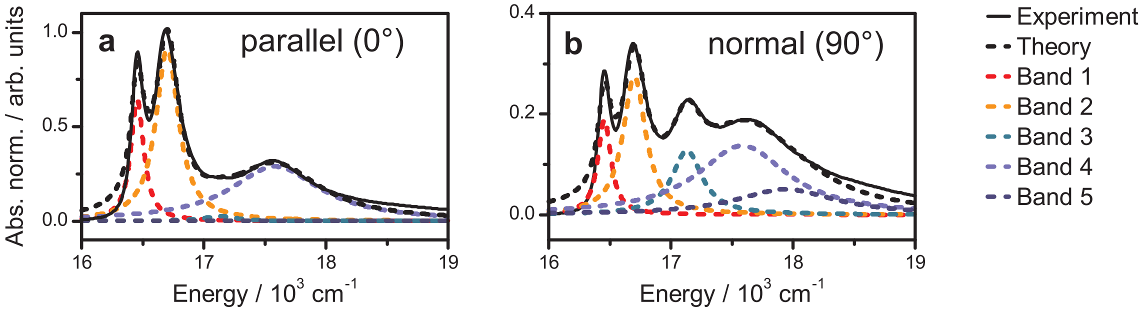

Supplementary Figure 3. **Absorption spectrum of C8O3**. **a**, Absorption spectrum with light polarized parallel to the longitudinal axis of C8O3. Experimental and theoretical results are shown as a black solid line and a black dashed line, respectively. Theoretical results were modeled by a sum of Lorentzian functions, which describe bands 1-5 of C8O3. Each Lorentzian function is shown as a colored dashed line. **b**, Absorption spectrum with light polarized normal to the longitudinal axis of C8O3. Note that the vertical scales in **a** and **b** are different.

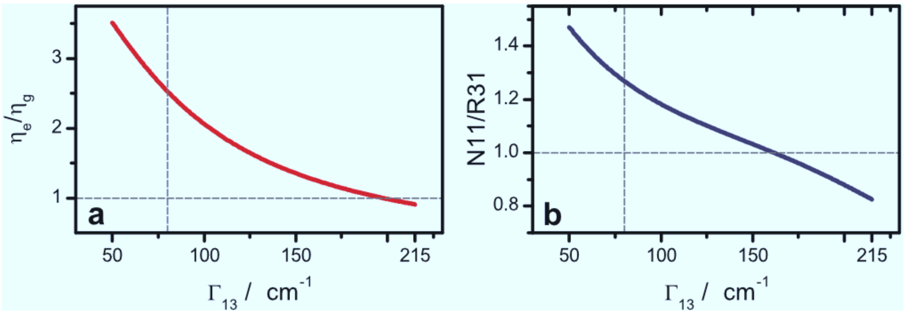

Supplementary Figure 4. **Long-lived beating signals in N11 and R31**. **a**, The ratio $|\eta_e/\eta_g|$ between the contributions of the vibronic and vibrational coherences to the long-lived beating signal in R31. For the experimentally estimated value of Γ_{13}, marked by a vertical dashed line, the contribution of the vibronic coherence is greater than the vibrational coherence. **b**, The ratio $\Gamma_{g3}(\Gamma_{g1}|\eta_e - \eta_g|)^{-1}$ between the amplitudes of the long-lived beating signals in N11 ($\propto \Gamma_{g1}^{-2}$) and R31 ($\propto \Gamma_{g3}^{-1}\Gamma_{g1}^{-1}|\eta_e - \eta_g|$). In both **a** and **b**, we take the values of the parameters estimated from experimental results. According to Eq. (19), $\hbar(\Gamma_{g1} + \Gamma_{g3}) \approx 215\,\mathrm{cm}^{-1}$ is the theoretical upper bound for Γ_{13}.

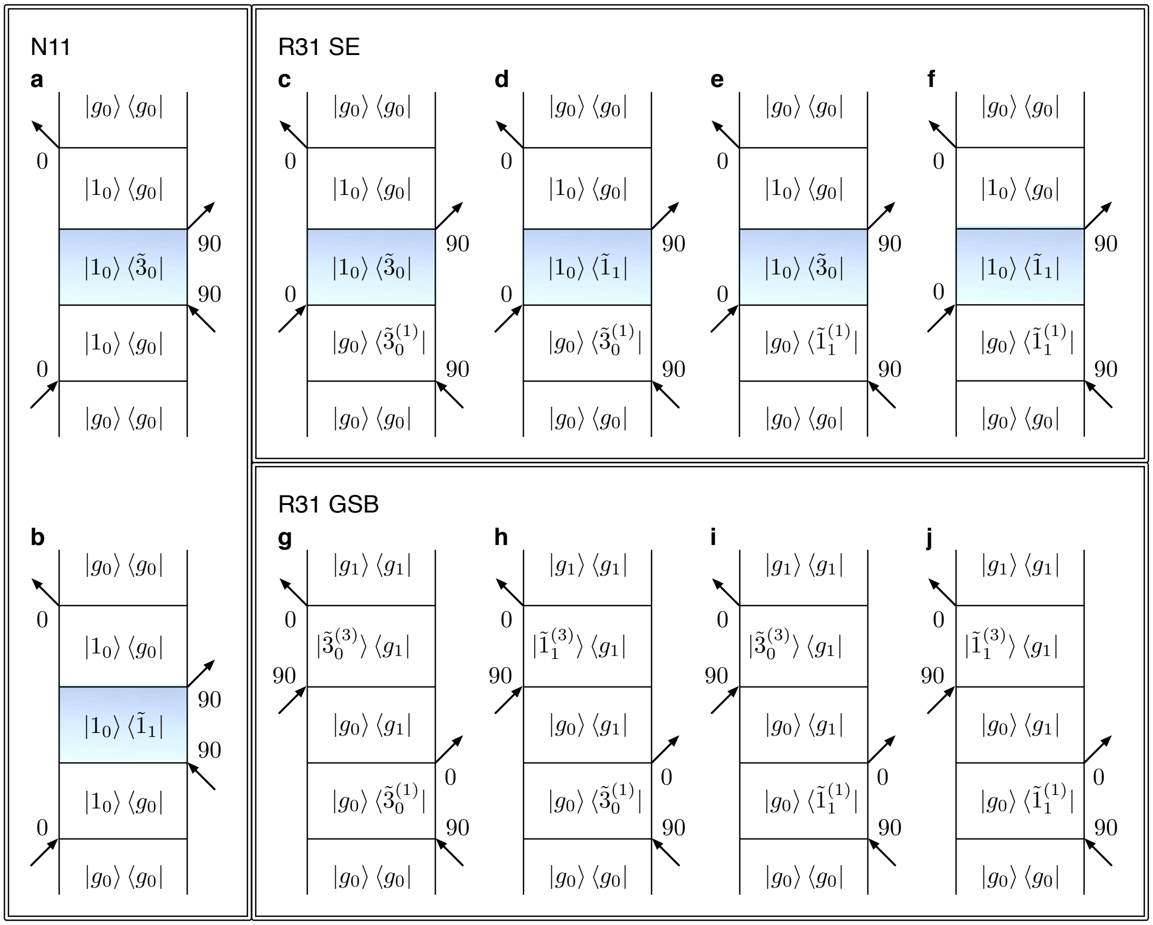

Supplementary Figure 5. **Feynman diagrams contributing to the beating signals in N11 and R31 represented in vibronic eigenbasis**. **a,b**, The stimulated emission diagrams contributing to the beating signals in N11. **c-f**, The stimulated emission diagrams contributing to the beating signals in R31. **g-j**, The ground state bleaching diagrams contributing to the beating signals in R31.

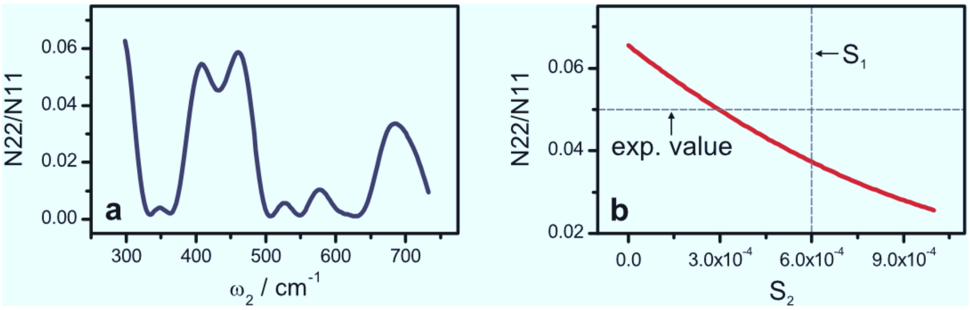

Supplementary Figure 6. **The relative amplitude of N22 and N11**. **a**, The absolute square of the Fourier transform of the beating signal in N22 as a function of ω_2, which is normalized to the amplitude of N11 at $\hbar\omega_2 \approx 705\,\mathrm{cm}^{-1}$. **b**, Theoretical results of the ratio between N22 and N11 are displayed as a function of the Huang-Rhys factor S_2. The Huang-Rhys factor $S_1 = 0.0006$ of the vibrational mode with frequency $\hbar\nu_1 \approx 668\,\mathrm{cm}^{-1}$ is marked by a vertical dashed line.

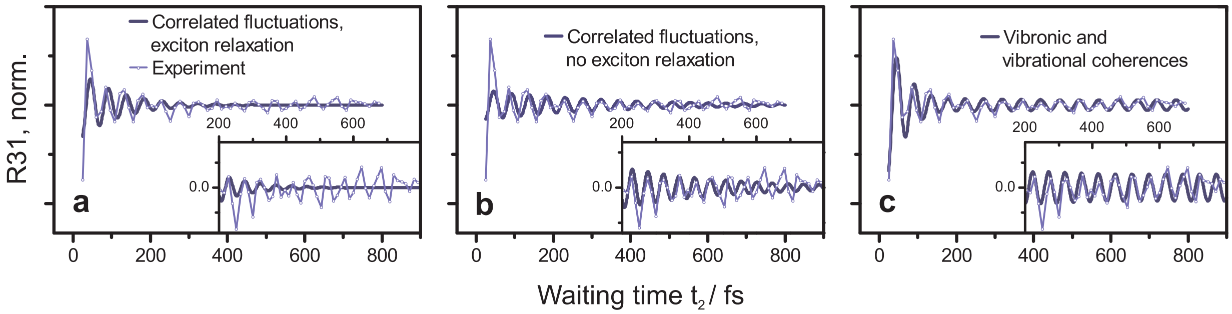

Supplementary Figure 7. **Correlated fluctuation model**. **a**, Simulated beating signal for R31 in the presence of correlated fluctuations (without quasi-resonant vibrations). The modeled curve is shown as a solid blue line and experimental results are shown as a light blue line. Here we take the lowest decoherence rate $\Gamma_{13} \approx (100\,\mathrm{fs})^{-1}$ of the inter-exciton coherence allowed by correlated fluctuations, constrained by experimentally determined population transfer rates from band 3 to bands 1 and 2. As shown in the inset, correlated fluctuations cannot explain the experimentally measured long-lived beating signal in R31. **b**, Simulated beating signal for R31 in the absence of the exciton relaxation, shown as a solid blue line. The correlated fluctuation model predicts the lowest decoherence rate $\Gamma_{13} \approx (303\,\mathrm{fs})^{-1}$ of the inter-exciton coherence when there is no exciton relaxation, even though this condition is not satisfied for C8O3. Nevertheless, the correlated fluctuation model cannot explain the experimentally measured long-lived beating signal in R31, which persist beyond $t_2 \approx 800\,\mathrm{fs}$, as shown in an inset. **c**, Simulated beating signal for R31 in the presence of a vibrational mode with frequency $\hbar\nu_1 \approx 668\,\mathrm{cm}^{-1}$, which is quasi-resonant with the exciton energy splitting $\Delta\Omega_{31}$ between bands 1 and 3. Here the vibronic and vibrational coherences induce a long-lived beating signal in good agreement with the experimental results. The root-mean-square deviation (RMSD) of the experimental results and theoretical prediction in **a**, **b** and **c** is 0.74, 0.86 and 0.59, respectively. We note that the correlated fluctuation model can also not explain the long-lasting beating signal in N11 (not shown).

Supplementary Notes:

Supplementary Note 1: Experiment

1. Sample preparation

The monomer, tetrachlorobenzimidacarbocyanine chromophore with two attached hydrophobic octyl groups (FEW-Chemicals, Wolfen, Germany) was dissolved in 10^{-2} M NaOH solution to achieve a concentration of 10^{-4} M. The solution was then stirred in the dark for several hours. Subsequently, polyvinyl alcohol (PVA) of molecular weight ~130000 was added in 1:10 w/w ratio (monomer:PVA) to slow down the formation of aggregate bundles during the storage of dye solutions. Moreover, the adsorbed PVA chains[1] obviously prevent the reassembly of double-layered into single-layered tubes upon bundling. This effect was observed recently for another derivative[2] (C8S3) of the present dye. The individual tubular aggregates degrade in that case into single-layered tubes, which is accompanied by a dramatic change of absorption spectra. Similar effects were not observed for C8O3 when PVA is present. In particular, the aggregate solutions prepared in the described way were stable for approximately ten days when stirred continuously. Without stirring the spectral signature of the double-layered tubes retained even after 12 weeks of storage[3]. For 2D experiments, we additionally diluted the sample with 10^{-2} M NaOH to obtain optical density below 0.3 at 598 nm at a path length of 200 μm.

A total sample volume of approximately 10 ml was circulated through the U-shaped wire-guided jet[4] by a peristaltic pump (Masterflex C/L) with a flow speed optimized for film stability. Solvent evaporated from the recollecting container was refilled every 4 hours during the course of 13 hour measurement.

2. Data acquisition

Passively stabilized 2D spectroscopy was described in detail elsewhere[5]. Briefly, a home-built non-collinear optical parametric amplifier (NOPA) seeded by 180 fs pulses at 1030 nm from PHAROS (Light Conversion Ltd) was tuned to generate ~16 fs pulses (80 nm full width at half maximum) centered at 580 nm. The NOPA output was split into four pulses and arranged in the so-called boxcar geometry. Waiting time t_2 was controlled by a mechanical translation stage (PI), whereas coherence time t_1 was scanned by inserting a pair of fused silica wedges into the first two pulses. All four pulses were focused and overlapped in the sample. The first three generated a third order nonlinear optical response which is emitted in the photon echo phase-matched direction. This signal was heterodyned with an attenuated fourth pulse, called local oscillator (LO). The resulting interference pattern was spectrally resolved and detected by a CCD camera (PIXIS, Princeton Instruments). Most of the scatter was eliminated by the double-frequency lock-in modulation of the first two pulses[6]. The polarization of each pulse was controlled by the combination of $\lambda/4$ wave plates and wire grid polarizers (contrast ratio > 800). The accuracy of the polarization angle was estimated to be $\pm 1°$, where the unwanted signals were typically suppressed by a factor of ~80 for the selected polarization sequence.

To prevent degradation of the sample, the power and repetition rate of the laser were set to 200 pJ/pulse and 40 kHz, respectively. Spectral resolution of ~35 cm^{-1} for the detection frequency ω_3 was determined by the grating, the number of CCD pixels and Fourier filtering of the signal during the standard analysis procedure. Coherence time was scanned from −300 fs to 384 fs in 1.5 fs steps, providing ~43 cm^{-1} spectral resolution of absorption frequency ω_1. Waiting time steps of 12 fs were sufficient to resolve oscillatory features up to 1350 cm^{-1} with ~35 cm^{-1} resolution along ω_2.

3. Polarization-controlled 2D-ES

The strength of 2D signals is determined by the scalar products of molecular transition dipole moments and pulse polarizations. To take advantage of i) the preferential orientation of the J-aggregate (from here on referred to as C8O3) along the flow direction of the jet and ii) mutually perpendicular transition dipole moments of bands 1(2) and 3 of C8O3, we designed a polarization scheme selective for interband coherences. This is similar to the case of an isotropic sample discussed both theoretically[7] and experimentally[8,9]. In the presented experiments, the polarization scheme for rephasing signals reads $(90, 0, 90, 0)$ for beams 1-4, respectively. The first and third pulses, polarized orthogonal (90) to the jet's flow direction, interact with bands 1-3. The second pulse, polarized parallel (0) to the jet's flow direction, interacts preferentially with bands 1 and 2, due to the negligible transition dipole moment of band 3 along this direction. The polarization scheme for non-rephasing spectra reads $(0, 90, 90, 0)$, as the ordering of the first two pulses is reversed. These polarization schemes restrict oscillatory signals induced by interband coherences to the lower cross peak in rephasing spectra (R31) and the lower diagonal peak in non-rephasing spectra (N11), as shown in Figures 2 and 3 of the main text. Non-oscillatory 2D signals were subtracted prior to Fourier transformation $t_2 \to \omega_2$.

The polarization-controlled 2D spectra were phased to pump-probe data where pump and probe pulses were polarized in parallel. This procedure is not rigorously correct because in polarization-controlled 2D-ES the first two pulses have different polarization directions while in pump-probe the first two interactions derive from the same pump pulse which naturally means parallel interactions. In other words, the projection slice theorem is strictly speaking not valid for the experiments presented here[10]. Despite this discrepancy, one can still satisfactorily phase polarization-controlled 2D spectra to all-parallel pump-probe

as shown in Supplementary Figure 1. One explanation of this is leakage of the much stronger all-parallel signals through the crossed polarizers, meaning that the all-parallel signal still dominates the non-oscillatory part of the (90,0,90,0) 2D-signal. In this work, we decided to phase polarization-controlled 2D data to parallel pump-probe data. We note that the imperfection in phasing parameters only affects the lineshapes of the real and imaginary part of ω_2 maps, but preserves their amplitude-maps in both lineshape and magnitude. Hence, the difficulties in phasing polarization-controlled 2D spectra discussed above do not affect the conclusions drawn in the main part of the main text, which were based on ω_2 amplitude-maps. To this end, we found that arbitrary and large changes of the phasing parameters do not alter ω_2 amplitude-maps shown in Figure 2 of the main text (results not presented). It is noted that sophisticated phasing techniques based on heterodyned transient grating instead of pump-probe offer a correct method to phase crossed-polarization 2D signals[11].

Supplementary Note 2: Theory of Coherent Vibronic Coupling

1. A vibronic model for bands 1 and 3 of C8O3

In the following the vibronic model used to describe bands 1 and 3 of C8O3 and simulate 2D spectra is described. We consider coherent interaction of bands 1 and 3 with the intramolecular vibrational modes of frequency $\hbar\nu_1 \approx 668\,\mathrm{cm}^{-1}$, which is quasi-resonant with the exciton energy splitting between bands 1 and 3. The environmental noise induced by background phonons (a phonon bath) is modeled by a Markovian quantum master equation.

1-1. Hamiltonian

The electronic Hamiltonian of C8O3 that consists of a network of cyanine dye molecules is described by

$$H_e = \sum_{\alpha} \hbar E_\alpha \left|e_\alpha\right\rangle \left\langle e_\alpha\right| + \sum_{\alpha\neq\beta} \hbar J_{\alpha\beta} \left|e_\alpha\right\rangle \left\langle e_\beta\right| \tag{1}$$

$$= \sum_k \hbar\Omega_k \left|k\right\rangle \left\langle k\right|, \tag{2}$$

where $|e_\alpha\rangle$ represents the excited state of site α (or molecule α), E_α denotes the site energy including electronic and reorganization energies, and $J_{\alpha\beta}$ the electronic coupling between sites α and β. The diagonalization of the electronic Hamiltonian H_e gives rise to the exciton states $|k\rangle = \sum_\alpha |e_\alpha\rangle \langle e_\alpha|k\rangle$ associated with the exciton energies Ω_k, where bands 1 and 3 are denoted by $|1\rangle$ and $|3\rangle$, respectively: $\langle e_\alpha|k\rangle \in R$ for $E_\alpha, J_{\alpha\beta} \in R$.

The vibrational modes with frequency $\hbar\nu_1 \approx 668\,\mathrm{cm}^{-1}$ are described by a set of harmonic oscillators

$$H_v = \sum_\alpha \hbar\nu_1 a_\alpha^\dagger a_\alpha, \tag{3}$$

where $a_\alpha^\dagger$ and a_α represent the creation and annihilation operators, respectively, of the intramolecular vibrational mode of site α. The interaction between vibrations and the electronic excitation of molecules is modeled by

$$H_{e-v} = \sum_\alpha \hbar\nu_1 \sqrt{s_1} \left|e_\alpha\right\rangle \left\langle e_\alpha\right| (a_\alpha^\dagger + a_\alpha), \tag{4}$$

where s_1 denotes the Huang-Rhys factor of the vibrational modes. In the exciton basis $\{|k\rangle\}$, the interaction Hamiltonian H_{e-v} is represented by

$$H_{e-v} = \hbar\nu_1 \sqrt{s_1} \sum_{k,l} \left|k\right\rangle \left\langle l\right| \sum_\alpha \langle k|e_\alpha\rangle\langle e_\alpha|l\rangle (a_\alpha^\dagger + a_\alpha), \tag{5}$$

where the diagonal terms ($k = l$) lead to adiabatic surfaces in the electronic excited states, called vibrons, while the non-diagonal terms ($k \neq l$) induce coherent transition between different excitons mediated by exciton-vibrational couplings.

In this work, we are interested in the coherent interaction of bands 1 and 3 with the quasi-resonant vibrational modes of frequency ν_1, which is described by the following cross term $\tilde{H}_{e-v}$ in Eq. (5)

$$\tilde{H}_{e-v} = \hbar\nu_1 \sqrt{S_1}(\left|1\right\rangle \left\langle 3\right| + \left|3\right\rangle \left\langle 1\right|)(\tilde{a}_1^\dagger + \tilde{a}_1), \tag{6}$$

where $\tilde{a}_1 = N \sum_\alpha \langle 1|e_\alpha\rangle\langle e_\alpha|3\rangle\, a_\alpha$ describes an effective vibrational mode with frequency ν_1. Here N is introduced to normalize the effective vibrational mode, such that $[\tilde{a}_1, \tilde{a}_1^\dagger] = 1$, leading to an effective Huang-Rhys factor $S_1 = s_1/N^2$. This implies that for a

given Huang-Rhys factor s_1, the effective Huang-Rhys factor S_1 is increased as the spatial overlap $\langle 1|e_\alpha\rangle\langle e_\alpha|3\rangle$ between excitonic wavefunctions of bands 1 and 3 increases, leading to smaller N and larger $S_1 = s_1/N^2$. The effective Hamiltonian of bands 1 and 3 coupled to the effective vibrational mode is then described by $\tilde{H} = \tilde{H}_e + \tilde{H}_v + \tilde{H}_{e-v}$, where $\tilde{H}_e = \hbar\Omega_1 |1\rangle\langle 1| + \hbar\Omega_3 |3\rangle\langle 3|$ and $\tilde{H}_v = \hbar\nu_1\tilde{a}_1^\dagger\tilde{a}_1$. We note that the vibrational energy $\hbar\nu_1 \approx 668\,\mathrm{cm}^{-1}$ is higher than the thermal energy $k_BT \approx 208\,\mathrm{cm}^{-1}$ at room temperature $T = 300\,K$, implying that the thermal state of the vibrational mode is well approximated by its ground state. In addition, when exciton-vibrational couplings are sufficiently small, the light-induced vibrational excitation of overtones is negligible due to the small Franck-Condon factors. This is the case for C8O3, where N11 and R31 in 2D spectra can be well described within a subspace spanned by $\{|g_0\rangle, |g_1\rangle, |1_0\rangle, |1_1\rangle, |3_0\rangle\}$. Here, $|k_0\rangle$ and $|k_1\rangle$ denote the vibrational ground and first excited states of an electronic state $|k\rangle$, respectively, *i.e.* $(\tilde{H}_e+\tilde{H}_v)|k_l\rangle = \hbar(\Omega_k + l\nu_1)|k_l\rangle$, where $|g\rangle$ represents the electronic ground state with $\Omega_g = 0$. In this scenario, $\{|1_0\rangle, |3_0\rangle\}$ can be directly excited by light from the ground state $|g_0\rangle$, while $\{|g_1\rangle, |1_1\rangle\}$ has an extremely low transition probability due to small Franck-Condon factors. Nonetheless, $\{|g_1\rangle, |1_1\rangle\}$ can be populated through exciton-vibrational coupling $\nu_1\sqrt{S_1}$, leading to transition from $|3_0\rangle$ to $|1_1\rangle$, and subsequently to $|g_1\rangle$ via emission. The coherent transition between $|3_0\rangle$ and $|1_1\rangle$ requires resonance between vibrational frequency ν_1 and exciton energy splitting $\Delta\Omega_{31} = \Omega_3 - \Omega_1$ between bands 1 and 3, *i.e.* $\Delta\Omega_{31} \approx \nu_1$.

1-2. Decoherence

In addition to the coherent interaction of bands 1 and 3 with the effective vibrational mode $\tilde{a}_1$, we consider electronic decoherence induced by background phonons. We characterize the decoherence by two dynamical processes, i) the incoherent population transfer between excitons, called exciton relaxation, and ii) the pure dephasing noise that destroys electronic coherence without exciton population transfer. In addition, we consider iii) relaxation of the effective vibrational mode.

We assume that each cyanine dye molecule is coupled to an independent phonon bath. The Hamiltonian of the background phonons is given by $H_{\mathrm{ph}} = \sum_\xi \hbar\upsilon_\xi b_\xi^\dagger b_\xi$ with the interaction Hamiltonian $H_{e-\mathrm{ph}} = \sum_{\alpha,\xi}\hbar g_{\alpha\xi}|e_\alpha\rangle\langle e_\alpha|(b_\xi^\dagger + b_\xi)$ between molecules and phonons, where $b_\xi^\dagger$ and b_ξ denote the creation and annihilation operators, respectively, of a background phonon mode ξ. Here $g_{\alpha\xi}$ represents the exciton-phonon coupling between site α and phonon mode ξ, which satisfies $g_{\alpha\xi}g_{\beta\xi} = 0$ for all $\beta \neq \alpha$, implying that when site α is coupled to the phonon mode ξ with $g_{\alpha\xi} \neq 0$, all the other sites β are decoupled from the mode with $g_{\beta\xi} = 0$. For the sake of simplicity, we assume that there is no degeneracy in the exciton energies Ω_k, which leads to a relatively simple form of a Markovian quantum master equation. This condition is satisfied even if the exciton energies are close to degeneracy unless they are strictly degenerate, which is satisfied for bands 1 and 3 of our interest. The influence of the background phonons on the vibronic system consisting of bands 1 and 3 with the effective vibrational mode is then described by a Markovian quantum master equation[12]

$$\frac{d}{dt}\rho(t) = -\frac{i}{\hbar}[\tilde{H}, \rho(t)] + \mathcal{D}_r[\rho(t)] + \mathcal{D}_d[\rho(t)] + \mathcal{D}_v[\rho(t)], \tag{7}$$

where $\rho(t)$ denotes the reduced vibronic state, while $\mathcal{D}_r[\rho(t)]$, $\mathcal{D}_d[\rho(t)]$ and $\mathcal{D}_v[\rho(t)]$ describe exciton relaxation, pure dephasing noise and relaxation of the effective vibrational mode, respectively.

i. Exciton relaxation

Here $\mathcal{D}_r[\rho(t)]$ describes exciton relaxation

$$\mathcal{D}_r[\rho(t)] = \sum_{\omega\neq 0}\sum_\alpha \gamma_{\alpha\alpha}(\omega)\left(A_\alpha(\omega)\rho(t)A_\alpha^\dagger(\omega) - \frac{1}{2}\{A_\alpha^\dagger(\omega)A_\alpha(\omega), \rho(t)\}\right), \tag{8}$$

with $\Delta\Omega_{kl} = \Omega_k - \Omega_l$ denoting the exciton energy splitting between $|k\rangle$ and $|l\rangle$, $A_\alpha(\omega) = \sum_{k,l}\delta(\omega, \Delta\Omega_{kl})\langle l|e_\alpha\rangle\langle e_\alpha|k\rangle|l\rangle\langle k|$ for $\omega \neq 0$, leading to incoherent transition from $|k\rangle$ to $|l\rangle$, where $\delta(i, j)$ denotes the Kronecker delta defined by $\delta(i, j) = 1$ if $i = j$ and $\delta(i, j) = 0$ otherwise. In Eq. (8), $\gamma_{\alpha\alpha}(\omega)$ is defined by

$$\gamma_{\alpha\alpha}(\omega) = 2\pi\mathcal{J}_\alpha(\omega)(n(\omega) + 1), \tag{9}$$

with $n(\omega) = (\exp(\hbar\omega/k_BT) - 1)^{-1}$ representing the Bose-Einstein distribution function at temperature T, $\mathcal{J}_\alpha(\omega)$ is the spectral density of site α defined by $\mathcal{J}_\alpha(\omega) = \sum_\xi g_{\alpha\xi}^2\delta(\omega - \upsilon_\xi)$ if $\omega \geq 0$ and $\mathcal{J}_\alpha(\omega) = -\mathcal{J}_\alpha(-\omega)$ otherwise. Here $\delta(x)$ represents the Dirac delta function defined by $\delta(x) \to \infty$ if $x = 0$ and $\delta(x) = 0$ otherwise with $\int_{-\infty}^{\infty} dx\delta(x) = 1$.

ii. Pure dephasing noise

$\mathcal{D}_d[\rho(t)]$ in Eq. (7) describes the pure dephasing noise

$$\mathcal{D}_d[\rho(t)] = \sum_\alpha \gamma_{\alpha\alpha}(0)\left(A_\alpha(0)\rho(t)A_\alpha^\dagger(0) - \frac{1}{2}\{A_\alpha^\dagger(0)A_\alpha(0), \rho(t)\}\right), \tag{10}$$

where $A_\alpha(0) = \sum_k |\langle k|e_\alpha\rangle|^2 |k\rangle\langle k|$ destroys electronic coherence without changing exciton populations defined by $\{\mathrm{Tr}[\langle k|\rho(t)|k\rangle]\}$.

By substituting electronic coherences $|g\rangle\langle 1|$, $|g\rangle\langle 3|$ and $|1\rangle\langle 3|$ to the dissipators $\mathcal{D}_r[\rho(t)]$ and $\mathcal{D}_d[\rho(t)]$ in Eqs. (8) and (10), one can obtain the following electronic decoherence rates Γ_{g1}, Γ_{g3} and Γ_{13} of the coherences $|g\rangle\langle 1|$, $|g\rangle\langle 3|$ and $|1\rangle\langle 3|$

$$\Gamma_{g1} = \frac{1}{2}\sum_{l\neq 1}\gamma_{1\to l} + \gamma_{g1}, \tag{11}$$

$$\Gamma_{g3} = \frac{1}{2}\sum_{l\neq 3}\gamma_{3\to l} + \gamma_{g3}, \tag{12}$$

$$\Gamma_{13} = \frac{1}{2}\sum_{l\neq 1}\gamma_{1\to l} + \frac{1}{2}\sum_{l\neq 3}\gamma_{3\to l} + \gamma_{13}, \tag{13}$$

where $\gamma_{k\to l}$ denotes the incoherent population transfer rate from band k to l

$$\gamma_{k\to l} = \sum_\alpha \gamma_{\alpha\alpha}(\Delta\Omega_{kl})\,|\langle l|e_\alpha\rangle\langle e_\alpha|k\rangle|^2 \geq 0, \tag{14}$$

while γ_{g1} and γ_{g3} represent the pure dephasing rates of the coherences $|g\rangle\langle 1|$ and $|g\rangle\langle 3|$, respectively,

$$\gamma_{g1} = \frac{1}{2}\sum_\alpha |\langle 1|e_\alpha\rangle|^2\,\gamma_{\alpha\alpha}(0)\,|\langle 1|e_\alpha\rangle|^2\,, \tag{15}$$

$$\gamma_{g3} = \frac{1}{2}\sum_\alpha |\langle 3|e_\alpha\rangle|^2\,\gamma_{\alpha\alpha}(0)\,|\langle 3|e_\alpha\rangle|^2\,, \tag{16}$$

and γ_{13} represents the pure dephasing rate of the inter-exciton coherence $|1\rangle\langle 3|$ between bands 1 and 3

$$\gamma_{13} = \frac{1}{2}\sum_\alpha \left(|\langle 1|e_\alpha\rangle|^2 - |\langle 3|e_\alpha\rangle|^2\right)\gamma_{\alpha\alpha}(0)\left(|\langle 1|e_\alpha\rangle|^2 - |\langle 3|e_\alpha\rangle|^2\right). \tag{17}$$

These results imply that the inter-exciton dephasing rate γ_{13} should be lower than the sum of the other dephasing rates γ_{g1} and γ_{g3} when there is a spatial overlap between excitonic wavefunctions of bands 1 and 3

$$\gamma_{13} = \gamma_{g1} + \gamma_{g3} - \sum_\alpha |\langle 1|e_\alpha\rangle|^2\,\gamma_{\alpha\alpha}(0)\,|\langle 3|e_\alpha\rangle|^2 \leq \gamma_{g1} + \gamma_{g3}, \tag{18}$$

with $\gamma_{\alpha\alpha}(0) \geq 0$ for all α, the equality $\gamma_{13} = \gamma_{g1} + \gamma_{g3}$ holds if and only if there is no spatial overlap between excitonic wavefunctions, *i.e.* $|\langle 1|e_\alpha\rangle|^2 |\langle 3|e_\alpha\rangle|^2 = 0$ for all α, or the spectral densities $\mathcal{J}_\alpha(\omega)$ of the molecules shared by bands 1 and 3 do not induce pure dephasing noise by $\gamma_{\alpha\alpha}(0) = 0$ for all sites α satisfying $|\langle 1|e_\alpha\rangle|^2 |\langle 3|e_\alpha\rangle|^2 \neq 0$. This implies that even if each molecule is coupled to an independent phonon bath, the spatial overlap between excitonic wavefunctions can reduce the inter-exciton dephasing rate γ_{13}. Here the independent phonon baths of the molecules shared by excitons effectively form a common phonon bath coupled to both excitons, leading to a partial dephasing-free subspace. For instance, if bands 1 and 3 have perfect spatial overlap, *i.e.* $|\langle 1|e_\alpha\rangle|^2 = |\langle 3|e_\alpha\rangle|^2$ for all α, while the orthogonality between them is satisfied by the phases of $\langle 1|e_\alpha\rangle\langle e_\alpha|3\rangle$, *i.e.* $\langle 1|3\rangle = \sum_\alpha \langle 1|e_\alpha\rangle\langle e_\alpha|3\rangle = 0$, the inter-exciton dephasing rate γ_{13} will become zero, as each $A_\alpha(0) = \sum_k |\langle k|e_\alpha\rangle|^2 |k\rangle\langle k| = |\langle 1|e_\alpha\rangle|^2 (|1\rangle\langle 1| + |3\rangle\langle 3|) + \sum_{k\neq 1,3} |\langle k|e_\alpha\rangle|^2 |k\rangle\langle k|$ forms a dephasing-free subspace $|1\rangle\langle 1| + |3\rangle\langle 3|$ of bands 1 and 3. Since band 1 is localized on the inner layer of C8O3, while band 3 is delocalized on both the inner and outer layers[13], there is a partial spatial overlap between excitonic wavefunctions, leading to $\gamma_{13} < \gamma_{g1} + \gamma_{g3}$. The spatial overlap is also required for a non-zero value of the effective Huang-Rhys factor S_1, which is responsible for the long-lived beating signals observed in the experiment, as will be discussed later.

In addition, the inter-exciton dephasing rate γ_{13} has a non-zero lower bound when the dephasing rates γ_{g1} and γ_{g3} are different in magnitude. The dephasing rates in Eqs. (15)-(17) can be expressed as $\gamma_{g1} = |\vec{v}_1|^2$, $\gamma_{g3} = |\vec{v}_3|^2$ and $\gamma_{13} = |\vec{v}_1 - \vec{v}_3|^2$ with the real vectors $\vec{v}_k$ defined by $\vec{v}_k = 2^{-1/2}\hat{\gamma}^{1/2}\vec{w}_k$, where $\vec{w}_k$ is a real vector with elements $|\langle k|e_\alpha\rangle|^2 \geq 0$ representing the delocalization of an exciton state $|k\rangle$ in the site basis $\{|e_\alpha\rangle\}$, while $\hat{\gamma}$ is a diagonalized matrix with elements $\gamma_{\alpha\alpha}(0) \geq 0$, leading to a positive matrix $\hat{\gamma}^{1/2}$ defined by $\hat{\gamma} = \hat{\gamma}^{1/2}\hat{\gamma}^{1/2}$. From the triangle inequality, $|\vec{v}_1 - \vec{v}_3| + |\vec{v}_3| \geq |\vec{v}_1|$ and $|\vec{v}_1 - \vec{v}_3| + |\vec{v}_1| \geq |\vec{v}_3|$, the inter-exciton dephasing rate γ_{13} is bounded from below by $(\sqrt{\gamma_{g1}} - \sqrt{\gamma_{g3}})^2$, leading to $(\sqrt{\gamma_{g1}} - \sqrt{\gamma_{g3}})^2 \leq \gamma_{13} < \gamma_{g1} + \gamma_{g3}$. Therefore, the electronic decoherence rate Γ_{13} of the inter-exciton coherence $|1\rangle\langle 3|$ is constrained by

$$\frac{1}{2}\sum_{l\neq 1}\gamma_{1\to l} + \frac{1}{2}\sum_{l\neq 3}\gamma_{3\to l} + \left[\left(\Gamma_{g1} - \frac{1}{2}\sum_{l\neq 1}\gamma_{1\to l}\right)^{1/2} - \left(\Gamma_{g3} - \frac{1}{2}\sum_{l\neq 3}\gamma_{3\to l}\right)^{1/2}\right]^2 \leq \Gamma_{13} < \Gamma_{g1} + \Gamma_{g3}, \tag{19}$$

with $\gamma_{gk} = \Gamma_{gk} - \frac{1}{2}\sum_{l\neq k}\gamma_{k\to l}$ from Eqs. (11) and (12). Here the population transfer rates ($\gamma_{1\to l}$ and $\gamma_{3\to l}$) and electronic decoherence rates (Γ_{g1} and Γ_{g3}) can be estimated using experimentally measured 2D spectra, which will be discussed later.

iii. Relaxation of quasi-resonant vibrations

Finally, $\mathcal{D}_v[\rho(t)]$ in Eq. (7) describes the relaxation of the effective vibrational mode

$$\mathcal{D}_v[\rho(t)] = \gamma_v(n(\nu_1)+1)\left(2\tilde{a}_1\rho(t)\tilde{a}_1^\dagger - \{\tilde{a}_1^\dagger\tilde{a}_1, \rho(t)\}\right) + \gamma_v n(\nu_1)\left(2\tilde{a}_1^\dagger\rho(t)\tilde{a}_1 - \{\tilde{a}_1\tilde{a}_1^\dagger, \rho(t)\}\right). \tag{20}$$

Since $n(\nu_1) \approx 0.04$ at room temperature $T = 300\,K$ due to the high vibrational energy $\hbar\nu_1 \gg k_B T$, Eq. (20) can be reduced to

$$\mathcal{D}_v[\rho(t)] \approx \gamma_v\left(2\tilde{a}_1\rho(t)\tilde{a}_1^\dagger - \{\tilde{a}_1^\dagger\tilde{a}_1, \rho(t)\}\right), \tag{21}$$

which describes the dissipation of the vibrational mode with the rate of γ_v.

1-3. The response function for N11

Here we derive the response function for the beating signals in N11, which is a diagonal peak in non-rephasing spectra centered at $(\omega_1, \omega_3) \approx (\Omega_1, \Omega_1)$.

In Supplementary Figure 2a, the Feynman diagram contributing to the beating signals in N11 after the employed (0, 90, 90, 0) excitation is displayed. As the thermal state of the effective vibrational mode at room temperature is well approximated by its ground state ($\hbar\nu_1 \gg k_B T$), the initial state of the vibronic system is given by $|g_0\rangle\langle g_0|$. After excitation to $|1_0\rangle\langle g_0|$ by the first pulse, the dynamics of $|1_0\rangle\langle g_0|$ during coherence time t_1 is governed by a time evolution super-operator $\mathcal{U}(t_1)$ determined by the quantum master equation in Eq. (7)

$$\mathcal{U}(t_1)\,|1_0\rangle\langle g_0| = e^{(-i\Omega_1-\Gamma_{g1})t_1}\,|1_0\rangle\langle g_0|, \tag{22}$$

for which the Fourier transform is given by

$$\int_0^\infty dt_1 e^{i\omega_1 t_1}\mathcal{U}(t_1)\,|1_0\rangle\langle g_0| = -\frac{1}{i(\omega_1-\Omega_1)-\Gamma_{g1}}\,|1_0\rangle\langle g_0|, \tag{23}$$

where the prefactor $-(i(\omega_1-\Omega_1)-\Gamma_{g1})^{-1}$ determines the lineshape of N11 along the ω_1-axis, which is centered at $\omega_1 = \Omega_1$ with a linewidth of $2\Gamma_{g1}$. By the second pulse, $|1_0\rangle\langle g_0|$ becomes $|1_0\rangle\langle 3_0|$, which evolves during waiting time t_2 into a mixture of $|1_0\rangle\langle 3_0|$ and $|1_0\rangle\langle 1_1|$, mediated by exciton-vibrational coupling, scaling with $\nu_1\sqrt{S_1}$. The time evolution of $|1_0\rangle\langle 3_0|$ is formally expressed as

$$\mathcal{U}(t_2)\,|1_0\rangle\langle 3_0| = |1_0\rangle\langle 3_0|\,e^{K t_2} = f(t_2)\,|1_0\rangle\langle 3_0| + g(t_2)\,|1_0\rangle\langle 1_1|, \tag{24}$$

where K is a non-Hermitian operator describing both the Hamiltonian dynamics and decoherence

$$K = (i\Delta\Omega_{31}-\Gamma_{13})\,|3_0\rangle\langle 3_0| + (i\nu_1-\gamma_v)\,|1_1\rangle\langle 1_1| + i\nu_1\sqrt{S_1}(|3_0\rangle\langle 1_1| + |1_1\rangle\langle 3_0|). \tag{25}$$

Here we evaluate $f(t_2)$ in Eq. (24), which describes the case that $|1_0\rangle\langle 3_0|$ becomes $|1_0\rangle\langle g_0|$ by the third pulse, as shown in Supplementary Figure 2a. By diagonalizing the non-Hermitian operator K, one can show that $f(t_2)$ is given by

$$f(t_2) = \sum_{k=1}^{2}\frac{1}{2}\left(1+(-1)^k\frac{x-y}{\sqrt{(x-y)^2+4z^2}}\right)\exp\left[\frac{1}{2}\left(x+y+(-1)^k\sqrt{(x-y)^2+4z^2}\right)t_2\right], \tag{26}$$

where $x = i\Delta\Omega_{31}-\Gamma_{13}$, $y = i\nu_1-\gamma_v$ and $z = i\nu_1\sqrt{S_1}$. Finally, $|1_0\rangle\langle g_0|$ evolves during rephasing time t_3

$$\mathcal{U}(t_3)\,|1_0\rangle\langle g_0| = e^{(-i\Omega_1-\Gamma_{g1})t_3}\,|1_0\rangle\langle g_0|, \tag{27}$$

for which the Fourier transform leads to the lineshape $-(i(\omega_3-\Omega_1)-\Gamma_{g1})^{-1}$ of N11 along the ω_3-axis. Therefore, the response function for N11 is given by

$$R_{1g}(\omega_1, t_2, \omega_3) = \mu_{1p}^2\mu_{3n}^2\frac{1}{i(\omega_1-\Omega_1)-\Gamma_{g1}}\frac{1}{i(\omega_3-\Omega_1)-\Gamma_{g1}}f(t_2), \tag{28}$$

where μ_{1p} denotes the transition dipole moment of band 1 for light polarized parallel to the longitudinal axis of C8O3, while μ_{3n} represents the transition dipole moment of band 3 for light polarized normal to the axis. This is due to the (0, 90, 90, 0)

polarization scheme employed for measuring non-rephasing spectra in the experiment, as schematically shown in Supplementary Figure 2a. It is notable that all the Feynman diagrams in Supplementary Figures 2a-e can be induced by $(90, 90, 90, 90)$ excitation where all the pulses are polarized normal to the longitudinal axis: band 1 can be excited or de-excited by both 0 and 90 polarizations, although with higher efficiency for light polarized at 0. For $(90, 90, 90, 90)$ excitation, the overall dipole strength $\mu_{1p}^2\mu_{3n}^2$ in Eq. (28) is decreased to $\mu_{1n}^2\mu_{3n}^2$ with $\mu_{1p}^2 > \mu_{1n}^2$, as band 1 is mainly polarized along the longitudinal axis of C8O3, as shown in the linear dichroism spectrum in Figure 1 of the main text. This implies that the $(0, 90, 90, 0)$ polarization scheme for non-rephasing spectra enhances the signal-to-noise ratio when compared to the $(90, 90, 90, 90)$ excitation. Similarly, the signal-to-noise ratio of rephasing spectra is enhanced by $(90, 0, 90, 0)$ excitation.

The lineshape function $(i(\omega_1 - \Omega_1) - \Gamma_{g1})^{-1}(i(\omega_3 - \Omega_1) - \Gamma_{g1})^{-1}$ in Eq. (28) shows that N11 is centered at $(\omega_1, \omega_3) = (\Omega_1, \Omega_1)$ with a symmetric linewidth $2\Gamma_{g1}$ along ω_1- and ω_3-axes. When $(\omega_1, \omega_3) = (\Omega_1, \Omega_1)$, the lineshape function is reduced to Γ_{g1}^{-2}, implying that the amplitude of the N11 peak is proportional to Γ_{g1}^{-2}, which is decreased as the linewidth $2\Gamma_{g1}$ increases. The time-dependent term $f(t_2)$ in Eq. (28) describes the evolution of N11 during waiting time t_2. In the absence of the exciton-vibrational coupling ($S_1 = 0$), $f(t_2)$ is reduced to

$$f(t_2)|_{S_1=0} = e^{(i\Delta\Omega_{31} - \Gamma_{13})t_2}, \tag{29}$$

implying that the coherence $|1_0\rangle\langle 3_0|$ oscillates with the frequency of the exciton energy splitting $\Delta\Omega_{31}$ and decays with the electronic decoherence rate Γ_{13}. Conversely, in the presence of the exciton-vibrational coupling ($S_1 > 0$), $f(t_2)$ is expressed as

$$f(t_2) = \frac{1}{2}\left(1 + \frac{x-y}{\sqrt{(x-y)^2 + 4z^2}}\right)e^{[i(\Delta\Omega_{31}+\delta\omega) - \Gamma_{13} + \delta\gamma]t_2} + \frac{1}{2}\left(1 - \frac{x-y}{\sqrt{(x-y)^2 + 4z^2}}\right)e^{[i(\nu_1 - \delta\omega) - \gamma_\nu - \delta\gamma]t_2}, \tag{30}$$

where $i\delta\omega + \delta\gamma = 2^{-1}[\sqrt{(x-y)^2 + 4z^2} - (x-y)]$, which satisfies $\delta\omega > 0$ and $\delta\gamma > 0$ for $\Delta\Omega_{31} > \nu_1$ and $\Gamma_{13} > \gamma_\nu$, which is the case for C8O3. There are several notable features that result from the vibronic coupling evident in Eq. (30). i) The first term, proportional to $e^{[i(\Delta\Omega_{31}+\delta\omega)-\Gamma_{13}+\delta\gamma]t_2}$, oscillates with a frequency of $\Delta\Omega_{31}' = \Delta\Omega_{31} + \delta\omega$, which is higher than the exciton energy splitting $\Delta\Omega_{31}$, and decays with the rate of $\Gamma_{13} - \delta\gamma$, which is lower than the electronic decoherence rate Γ_{13} shown in Eq. (29). These are the characteristics of the vibronic coherence $|1_0\rangle\langle\tilde{3}_0|$, where $\langle\tilde{3}_0|$ is one of the left eigenstates of K in the form of $\langle\tilde{3}_0| \propto \langle 3_0| + \xi\langle 1_1|$ with $|\xi| < 1$. The vibronic eigenstate $\langle\tilde{3}_0|$ has a higher energy-level than $\langle 3_0|$ due to the exciton-vibrational coupling, leading to $\Delta\Omega_{31}' > \Delta\Omega_{31}$ (see Figure 3a in the main text). Additionally, the amplitude of $|1_0\rangle\langle\tilde{3}_0|$ in $|1_0\rangle\langle 1_1|$ denoted by ξ leads to a longer lifetime than the coherence $|1_0\rangle\langle 3_0|$ that has no vibrational character, or in other words, the lifetime borrowing effect. ii) Conversely, the second term in Eq. (30), proportional to $e^{[i(\nu_1-\delta\omega)-\gamma_\nu-\delta\gamma]t_2}$, exhibits characteristics of the other vibronic coherence $|1_0\rangle\langle\tilde{1}_1|$, where $\langle\tilde{1}_1| \propto \langle 1_1| - \xi\langle 3_0|$ is the other left eigenstate of K. The second term oscillates with frequency $\nu_1' = \nu_1 - \delta\omega$, which is lower than the vibrational frequency ν_1 due to the exciton-vibrational coupling (see Figure 3a in the main text). It also decays with the rate of $\gamma_\nu + \delta\gamma$, which is higher than the vibrational decoherence rate γ_ν of $|1_0\rangle\langle 1_1|$ due to the amplitude of $|1_0\rangle\langle\tilde{1}_1|$ in $|1_0\rangle\langle 3_0|$ denoted by ξ. iii) We add that the vibronic states $\langle\tilde{3}_0| \propto \langle 3_0| + \xi\langle 1_1|$ and $\langle\tilde{1}_1| \propto \langle 1_1| - \xi\langle 3_0|$ are the eigenstates of the non-Hermitian operator K in Eq. (25) describing both Hamiltonian dynamics and decoherence, where ξ depends on the parameters of the Hamiltonian as well as decoherence rates. These states are different from the eigenstates of the Hamiltonian $\tilde{H}$, which do not depend on decoherence rates, and their difference becomes non-negligible when the electronic decoherence rate Γ_{13} is comparable to or larger than the exciton-vibrational coupling $\nu_1\sqrt{S_1}$, as is the case for C8O3.

By fitting experimental 2D spectra to the theoretical prediction of N11 and R31, which will be discussed later, we found that $\hbar\Delta\Omega_{31} \approx 720\,\mathrm{cm}^{-1}$, $\hbar\nu_1 \approx 668\,\mathrm{cm}^{-1}$, $\hbar\Gamma_{g1} \approx 65\,\mathrm{cm}^{-1}$, $\hbar\Gamma_{g3} \approx 150\,\mathrm{cm}^{-1}$, $\hbar\Gamma_{13} \approx 80\,\mathrm{cm}^{-1}$, $S_1 = 0.0006$ (*cf.* $\hbar\nu_1\sqrt{S_1} \approx 16\,\mathrm{cm}^{-1}$) and $\gamma_\nu \lesssim (1\,\mathrm{ps})^{-1}$. The estimated electronic decoherence rates Γ_{g1} and Γ_{g3} reproduce well the absorption spectrum of C8O3, as shown in Supplementary Figure 3, where experimental and theoretical results are shown as a black solid line and a black dashed line, respectively. The theoretical results were modeled by a sum of the Lorentzian functions with linewidths $2\Gamma_{gk}$ for $k \in \{1, 2, 3, 4, 5\}$, each of which describes the absorption of band k: each Lorentzian function is shown as a colored dashed line. The estimated values of the parameters lead to $\hbar\delta\omega \approx 1.6\,\mathrm{cm}^{-1}$ and $\hbar\delta\gamma \approx 2.1\,\mathrm{cm}^{-1}$, which are smaller than the experimental resolution of $\sim 40\,\mathrm{cm}^{-1}$. This implies that for the case of C8O3, we can approximate $\Delta\Omega_{31}'$ and ν_1' by $\Delta\Omega_{31}$ and ν_1, respectively, with $\delta\omega \approx 0$ and $\delta\gamma \approx 0$. More specifically, when the exciton-vibrational coupling is sufficiently small, such that $\nu_1\sqrt{S_1} < |i\Delta\nu_1 - \Gamma_{13}|$ with $\Delta\nu_1 = \Delta\Omega_{31} - \nu_1$, and the dissipation rate of the vibrational mode is negligible within the timescale of the total measurement time, *i.e.* $\gamma_\nu \approx 0$, the response function determining N11 in Eq. (28) is reduced to

$$R_{1g}(\omega_1, t_2, \omega_3) \approx \mu_{1p}^2\mu_{3n}^2 \frac{1}{i(\omega_1 - \Omega_1) - \Gamma_{g1}} \frac{1}{i(\omega_3 - \Omega_1) - \Gamma_{g1}} \left[e^{(i\Delta\Omega_{31} - \Gamma_{13})t_2}(1 - \epsilon_2^2) + e^{i\nu_1 t_2}\epsilon_2^2\right], \tag{31}$$

with ϵ_2 representing the degree of vibronic mixing during waiting time t_2

$$\epsilon_2 = i\nu_1\sqrt{S_1}(i\Delta\nu_1 - \Gamma_{13})^{-1}, \tag{32}$$

where the vibronic eigenstates $\langle\tilde{3}_0|$ and $\langle\tilde{1}_1|$ are approximated by $\langle\tilde{3}_0| \propto \langle 3_0| + \epsilon_2\langle 1_1|$ and $\langle\tilde{1}_1| \propto \langle 1_1| - \epsilon_2\langle 3_0|$, respectively, with $|\epsilon_2|^2 \ll 1$ (in the main text, ϵ_2 was denoted by ϵ for the sake of simplicity). It can be seen in Eq. (32) that $|\epsilon_2|$ increases as

the exciton-vibrational coupling $\nu_1\sqrt{S_1}$ increases or the detuning $|\Delta\nu_1| = |\Delta\Omega_{31} - \nu_1|$ between exciton splitting and vibrational frequency decreases. This implies that the vibronic mixing of the coherences $|1_0\rangle\langle 3_0|$ and $|1_0\rangle\langle 1_1|$ requires resonance between excitons and vibrations and induces the observed long-lived beating signal in N11. In this respect, when $|\epsilon_2|$ decreases as a result of a high electronic decoherence rate Γ_{13}, the coherence $|1_0\rangle\langle 3_0|$ generated by the second pulse (see Supplementary Figure 2a) will decohere too quickly and thereby suppressing the vibronic mixing of $|1_0\rangle\langle 3_0|$ and $|1_0\rangle\langle 1_1|$ during waiting time t_2, which in turn will suppress the long-lived beating signal in N11. This is related to the fact that ϵ_2 is proportional to the exciton-vibrational coupling $\nu_1\sqrt{S_1}$ and the amplitude of the long-lived component $e^{i\nu_1 t_2}$ in Eq. (31) is proportional to ϵ_2^2. As such, when $\nu_1\sqrt{S_1} < |i\Delta\nu_1 - \Gamma_{13}|$, the response function for N11 can be effectively described by two transitions between $|1_0\rangle\langle 3_0|$ and $|1_0\rangle\langle 1_1|$ during waiting time t_2, mediated by exciton-vibrational coupling $\nu_1\sqrt{S_1}$, *i.e.* $|1_0\rangle\langle 3_0| \to |1_0\rangle\langle 1_1| \to |1_0\rangle\langle 3_0|$, within the timescale of the electronic decoherence rate Γ_{13}, as shown in Supplementary Figure 2a. When the condition of $\nu_1\sqrt{S_1} < |i\Delta\nu_1 - \Gamma_{13}|$ is not satisfied, the response function for N11 is represented by $R_{1g}(\omega_1, t_2, \omega_3) = \sum_{n=0}^{\infty} h_n(\omega_1, t_2, \omega_3)(i\nu_1\sqrt{S_1})^{2n}$ with the higher order terms proportional to $(i\nu_1\sqrt{S_1})^{2n}$, which describe multiple transitions between $|1_0\rangle\langle 3_0|$ and $|1_0\rangle\langle 1_1|$ during t_2.

In summary, when $\nu_1\sqrt{S_1} < |i\Delta\nu_1 - \Gamma_{13}|$ and $\gamma_\nu \approx 0$, the response function for N11 at $(\omega_1, \omega_3) = (\Omega_1, \Omega_1)$ is given by

$$R_{1g}(t_2) \approx \mu_{1p}^2 \mu_{3n}^2 \Gamma_{g1}^{-2} \left[e^{(i\Delta\Omega_{31} - \Gamma_{13})t_2} + e^{i\nu_1 t_2}\epsilon_2^2 \right], \tag{33}$$

with ϵ_2 defined in Eq. (32). The lineshape of N11 is symmetric along ω_1- and ω_3-axes with a linewidth of $2\Gamma_{g1}$. These results are in line with the experimental observations shown in Figures 2 and 3 of the main text.

1-4. The response function for R31

Here we provide the response function for the beating signals in R31, which is the cross peak in the rephasing spectra centered at $(\omega_1, \omega_3) \approx (\Omega_3, \Omega_1)$. The response function for R31 can be derived using the same approach described above for N11. Here we provide the results without derivation. Supplementary Figures 2b-e show the Feynman diagrams contributing to the beating signals in R31. In Supplementary Figures 2b-d, the vibronic system is in the electronic excited states during t_2, while in Supplementary Figure 2e, the system is in the electronic ground state, each of which is called the stimulated emission (SE) and ground state bleaching (GSB) diagram, respectively.

When $\nu_1\sqrt{S_1} < |i\Delta\nu_1 - \Gamma_{13}|$, $\nu_1\sqrt{S_1} < \left|i\Delta\nu_1 + \Gamma_{g1} - \Gamma_{g3}\right|$ and $\gamma_\nu \approx 0$, which are satisfied for the case of C8O3, the contribution of the SE diagrams to R31 is approximated by

$$\begin{aligned} R_{2g}(\omega_1, t_2, \omega_3) \approx \mu_{1p}^2 \mu_{3n}^2 \Bigg\{ & \frac{1}{-i(\omega_1 - \Omega_3) - \Gamma_{g3}} \frac{1}{i(\omega_3 - \Omega_1) - \Gamma_{g1}} \left[e^{(i\Delta\Omega_{31} - \Gamma_{13})t_2}(1 - \epsilon_2^2) + e^{i\nu_1 t_2}\epsilon_2^2 \right] \\ & + \left(-\frac{1}{-i(\omega_1 - \Omega_3) - \Gamma_{g3}} + \frac{1}{-i(\omega_1 - \Omega_3 + \Delta\nu_1) - \Gamma_{g1}} \right) \frac{1}{i(\omega_3 - \Omega_1) - \Gamma_{g1}} \left(-e^{(i\Delta\Omega_{31} - \Gamma_{13})t_2} + e^{i\nu_1 t_2} \right) \epsilon_1 \epsilon_2 \\ & + \left(-\frac{1}{-i(\omega_1 - \Omega_3) - \Gamma_{g3}} + \frac{1}{-i(\omega_1 - \Omega_3 + \Delta\nu_1) - \Gamma_{g1}} \right) \frac{1}{i(\omega_3 - \Omega_1) - \Gamma_{g1}} e^{(i\Delta\Omega_{31} - \Gamma_{13})t_2}\epsilon_1^2 \Bigg\}, \end{aligned} \tag{34}$$

with ϵ_1 representing the degree of vibronic mixing during coherence time t_1

$$\epsilon_1 = i\nu_1\sqrt{S_1}(i\Delta\nu_1 + \Gamma_{g1} - \Gamma_{g3})^{-1}, \tag{35}$$

where $\Delta\Omega_{31}'$ and ν_1' are approximated by $\Delta\Omega_{31}$ and ν_1, respectively. More specifically, ϵ_1 is associated with the transition between $|g_0\rangle\langle 3_0|$ and $|g_0\rangle\langle 1_1|$ during t_1, while ϵ_2 is associated with the transition between $|1_0\rangle\langle 3_0|$ and $|1_0\rangle\langle 1_1|$ during t_2. In Eq. (34), the first term proportional to ϵ_2^2 describes the transition $|1_0\rangle\langle 3_0| \to |1_0\rangle\langle 1_1| \to |1_0\rangle\langle 3_0|$ during t_2 (see Supplementary Figure 2b), the second term proportional to $\epsilon_1\epsilon_2$ describes the transition $|g_0\rangle\langle 3_0| \to |g_0\rangle\langle 1_1|$ during t_1 and the subsequent transition $|1_0\rangle\langle 1_1| \to |1_0\rangle\langle 3_0|$ during t_2 (see Supplementary Figure 2c), and the last term proportional to ϵ_1^2 describes the transition $|g_0\rangle\langle 3_0| \to |g_0\rangle\langle 1_1| \to |g_0\rangle\langle 3_0|$ during t_1 (see Supplementary Figure 2d). In the second and last terms, the lineshape function along the ω_1-axis contains $(-i(\omega_1 - \Omega_3 + \Delta\nu_1) - \Gamma_{g1})^{-1}$, which describes the presence of a sub-peak centered at $\omega_1 = \Omega_3 - \Delta\nu_1 = \Omega_1 + \nu_1 < \Omega_3$ with a linewidth of $2\Gamma_{g1}$, which is induced by exciton-vibrational coupling. However, due to the condition of $|\epsilon_1|^2 \ll 1$ and $|\epsilon_2|^2 \ll 1$, the first term in Eq. (34) determines the overall lineshape of R31, which is given by $(-i(\omega_1 - \Omega_3) - \Gamma_{g3})^{-1}(i(\omega_3 - \Omega_1) - \Gamma_{g1})^{-1}$ that is centered at $(\omega_1, \omega_3) = (\Omega_3, \Omega_1)$ with the asymmetric linewidths of $2\Gamma_{g3}$ and $2\Gamma_{g1}$ along ω_1- and ω_3-axes, respectively.

The contribution of the GSB diagram to R31, with ground state coherence[14] during t_2, is given by

$$R_{3g}(\omega_1, t_2, \omega_3) \approx \left(\frac{1}{-i(\omega_1 - \Omega_3) - \Gamma_{g3}} - \frac{1}{-i(\omega_1 - \Omega_3 + \Delta\nu_1) - \Gamma_{g1}} \right) \left(\frac{1}{i(\omega_3 - \Omega_1 - \Delta\nu_1) - \Gamma_{g3}} - \frac{1}{i(\omega_3 - \Omega_1) - \Gamma_{g1}} \right) e^{i\nu_1 t_2}\epsilon_1\epsilon_3, \tag{36}$$

with ϵ_3 representing the vibronic mixing during t_3

$$\epsilon_3 = -i\nu_1\sqrt{S_1}(-i\Delta\nu_1 + \Gamma_{g1} - \Gamma_{g3})^{-1}, \tag{37}$$

which is associated with the transition $|3_0\rangle\langle g_1| \rightarrow |1_1\rangle\langle g_1|$ during t_3 shown in Supplementary Figure 2e. Here the vibrational frequency ν_1 in $e^{i\nu_1 t_2}$ stems from the vibrational coherence $|g_0\rangle\langle g_1|$ in the electronic ground-state manifold and not the result of the approximation $\delta\omega \approx 0$.

In summary, when $\nu_1\sqrt{S_1} < |i\Delta\nu_1 - \Gamma_{13}|$, $\nu_1\sqrt{S_1} < |i\Delta\nu_1 + \Gamma_{g1} - \Gamma_{g3}|$ and $\gamma_v \approx 0$, the response function for R31 at $(\omega_1, \omega_3) = (\Omega_3, \Omega_1)$ is given by

$$R_{2g}(t_2) + R_{3g}(t_2) \approx \mu_{1p}^2\mu_{3n}^2\Gamma_{g3}^{-1}\Gamma_{g1}^{-1}[e^{(i\Delta\Omega_{31}-\Gamma_{13})t_2} + e^{i\nu_1 t_2}\epsilon_2^2(\eta_e - \eta_g)], \tag{38}$$

where $\eta_e = (\Gamma_{g1} + \Gamma_{13})(\Gamma_{g1} + i\Delta\nu_1)^{-1}$ stems from the SE diagrams shown in Supplementary Figures 2b-d, while $\eta_g = (\Gamma_{13} - i\Delta\nu_1)^2(\Gamma_{g1} + i\Delta\nu_1)^{-1}(\Gamma_{g3} + i\Delta\nu_1)^{-1}$ originates from the GSB diagram shown in Supplementary Figure 2e. It is interesting to note that the origin of the long-lived oscillations at R31, whether predominantly vibrational or vibronic, depends upon the electronic decoherence rates $\{\Gamma_{g1}, \Gamma_{g3}, \Gamma_{13}\}$ and detuning $\Delta\nu_1 = \Delta\Omega_{31} - \nu_1$. In Supplementary Figure 4a, the ratio $|\eta_e/\eta_g|$ between the contributions of the vibronic and vibrational coherences to the long-lived beating signal in R31 is displayed as a function of the inter-exciton decoherence rate Γ_{13}, where $\{\Gamma_{g1}, \Gamma_{g3}, \Delta\nu_1\}$ are taken to be the values estimated from experimental results. Here $|\eta_e/\eta_g| > 1$ implies that the long-lived beating signal in R31 is dominated by the vibronic coherence $|1\rangle\langle\tilde{1}_1|$ in the electronic excited-state manifold. By fitting the experimentally measured beating signals in N11 and R31 to the theoretical model, we found that $\hbar\Gamma_{13} \approx 80\,\mathrm{cm}^{-1}$, which is marked by a vertical dashed line in Supplementary Figure 4a, where the contribution of the vibronic coherence is ~ 2.5 times greater than the vibrational coherence. These results imply that the long-lived beating signal in R31 is dominated by vibronic coherence, originating from electronic excited states. It is notable that the vibronic contribution outweighs the vibrational part for a wide range of Γ_{13}. This is mainly due to the fact that the vibronic mixing $\epsilon_2 \propto (i\Delta\nu_1 - \Gamma_{13})^{-1}$ during t_2 depends on the inter-exciton decoherence rate Γ_{13}, while the other vibronic mixings $\epsilon_1 \propto (i\Delta\nu_1 + \Gamma_{g1} - \Gamma_{g3})^{-1}$ and $\epsilon_3 \propto (-i\Delta\nu_1 + \Gamma_{g1} - \Gamma_{g3})^{-1}$ during t_1 and t_3 are independent of Γ_{13}. Considering that vibronic coherence depends on ϵ_2 (see Eq. (34) and Supplementary Figures 2b and c), while vibrational coherence depends on $\epsilon_1\epsilon_3$ (see Eq. (36) and Supplementary Figure 2e), the vibronic contribution is increased as Γ_{13} decreases. We note that these results are in line with the experimental observation that the amplitude of the long-lived beating signal in N11 is greater than that of R31 (see Figures 3b and c in the main text). In Supplementary Figure 4b, the ratio $\Gamma_{g3}(\Gamma_{g1}|\eta_e - \eta_g|)^{-1}$ between the amplitudes of the long-lived beating signals in N11 and R31 is displayed as a function of the inter-exciton decoherence rate Γ_{13}. Here the amplitude of the long-lived beating signal in N11 is greater than R31, *i.e.* $\Gamma_{g3}(\Gamma_{g1}|\eta_e - \eta_g|)^{-1} > 1$, for a range of Γ_{13} where the vibronic coherence dominates the long-lived beating signal in R31, as shown in Supplementary Figure 4a.

1-5. Numerical simulation of N11 and R31

So far the analytic form of the response functions for N11 and R31 were derived with the assumption that the vibronic system is well described within the subspace of the vibrational ground and first excited states, which is valid for a small Huang-Rhys factor S_1. To clarify the validity of this assumption, we performed numerical simulation of the beating signals in N11 and R31 with higher vibrational excited states, *i.e.* $\{|g_0\rangle, |g_1\rangle, \cdots, |g_n\rangle, |1_0\rangle, |1_1\rangle, \cdots, |1_n\rangle, |3_0\rangle, |3_1\rangle, \cdots, |3_n\rangle\}$ with $n \geq 1$. We found that the theoretical beating signals converge for $n \geq 1$ and the numerical results are well matched to the analytical results. Here the electronic decoherence was modeled by a convex combination of two effective dissipators, *i.e.* $p\mathcal{D}_1[\rho(t)] + (1-p)\mathcal{D}_2[\rho(t)]$ with $0 \leq p \leq 1$, where the dissipators are given by

$$\mathcal{D}_1[\rho(t)] = \Gamma_{g1}(2|1\rangle\langle 1|\rho(t)|1\rangle\langle 1| - \{|1\rangle\langle 1|, \rho(t)\}) + \Gamma_{g3}(2|3\rangle\langle 3|\rho(t)|3\rangle\langle 3| - \{|3\rangle\langle 3|, \rho(t)\}), \tag{39}$$

$$\mathcal{D}_2[\rho(t)] = 2\left(\sqrt{\Gamma_{g1}}|1\rangle\langle 1| + \sqrt{\Gamma_{g3}}|3\rangle\langle 3|\right)\rho(t)\left(\sqrt{\Gamma_{g1}}|1\rangle\langle 1| + \sqrt{\Gamma_{g3}}|3\rangle\langle 3|\right) - \{\Gamma_{g1}|1\rangle\langle 1| + \Gamma_{g3}|3\rangle\langle 3|, \rho(t)\}. \tag{40}$$

By substituting electronic coherences $|g\rangle\langle 1|$ and $|g\rangle\langle 3|$ to the dissipators, one can show that both $\mathcal{D}_1[\rho(t)]$ and $\mathcal{D}_2[\rho(t)]$ give rise to the same set of decoherence rates Γ_{g1} and Γ_{g3} for $|g\rangle\langle 1|$ and $|g\rangle\langle 3|$, respectively, implying that the decoherence rates of $|g\rangle\langle 1|$ and $|g\rangle\langle 3|$ are independent of the value of p in the convex combination. For $|1\rangle\langle 3|$, on the other hand, $\mathcal{D}_1[\rho(t)]$ and $\mathcal{D}_2[\rho(t)]$ lead to different decoherence rates $\Gamma_{g1} + \Gamma_{g3}$ and $(\sqrt{\Gamma_{g1}} - \sqrt{\Gamma_{g3}})^2$, respectively. This enables us to vary the inter-exciton decoherence rate Γ_{13} within a range of $(\sqrt{\Gamma_{g1}} - \sqrt{\Gamma_{g3}})^2 \leq \Gamma_{13} \leq \Gamma_{g1} + \Gamma_{g3}$ by changing the value of p in the convex combination. In addition to the electronic decoherence, the relaxation of the vibrational mode was modeled by Eq. (20) in the simulations. We found that Eq. (20) can be approximated by Eq. (21) due to the high vibrational frequency ($\hbar\nu_1 \gg k_B T$).

1-6. Feynman diagrams represented in vibronic eigenbasis

Here we provide the Feynman diagrams for N11 and R31 represented in the vibronic eigenbasis of the time evolution super-operator $\mathcal{U}(t)$, which are equivalent to the Feynman diagrams in the uncoupled state basis shown in Supplementary Figure 2.

For N11, the vibronic mixing ϵ_2 takes place during waiting time t_2 (*cf.* Supplementary Figure 2a), where the vibronic coherences responsible for the short-lived and long-lived beating signals in N11 are given by

$$|1_0\rangle\langle\tilde{3}_0| = (1+\epsilon_2^2)^{-1/2}(|1_0\rangle\langle 3_0| + \epsilon_2 |1_0\rangle\langle 1_1|), \tag{41}$$

$$|1_0\rangle\langle\tilde{1}_1| = (1+\epsilon_2^2)^{-1/2}(|1_0\rangle\langle 1_1| - \epsilon_2 |1_0\rangle\langle 3_0|), \tag{42}$$

respectively, where the vibronic eigenstates $\langle\tilde{3}_0| \propto \langle 3_0| + \epsilon_2\langle 1_1|$ and $\langle\tilde{1}_1| \propto \langle 1_1| - \epsilon_2\langle 3_0|$ are normalized by $(1+\epsilon_2^2)^{-1/2}$, not by $(1+|\epsilon_2|^2)^{-1/2}$, due to the biorthogonality of the eigenstates of the non-Hermitian operator K in Eq. (25). When the light-induced vibrational excitation of overtones, *i.e.* $\langle 1_1|$, is negligible due to the small Franck-Condon factors, the transition dipole moments of the vibronic eigenstates $\langle\tilde{3}_0|$ and $\langle\tilde{1}_1|$ are determined by their amplitudes in $\langle 3_0|$, each of which is given by $\mu_{3n}(1+\epsilon_2^2)^{-1/2}$ and $-\mu_{3n}(1+\epsilon_2^2)^{-1/2}\epsilon_2$, respectively. Here μ_{3n} denotes the transition dipole moment of $\langle 3_0|$. In the eigenbasis, the Feynman diagrams responsible for the short-lived and long-lived beating signals in N11 are described by Supplementary Figures 5a and b, respectively. Given that there are two transitions between $\langle g_0|$ and $\langle\tilde{3}_0|$ (and also between $\langle g_0|$ and $\langle\tilde{1}_1|$) by the second and third pulses, the square of the transition dipole moments of $\langle\tilde{3}_0|$ and $\langle\tilde{1}_1|$ is reflected in the response function, each of which is given by $\mu_{3n}^2(1+\epsilon_2^2)^{-1} \approx \mu_{3n}^2(1-\epsilon_2^2)$ and $\mu_{3n}^2\epsilon_2^2$, respectively. This is in line with the analytic form of the response function for N11 shown in Eq. (31).

For R31, on the other hand, vibronic mixing takes place during coherence, waiting and rephasing times (t_1, t_2, t_3, respectively, *cf.* Supplementary Figures 2b-e). The vibronic mixing ϵ_1 during coherence time t_1 leads to the vibronic eigenstates $\langle\tilde{3}_0^{(1)}| \propto \langle 3_0| + \epsilon_1\langle 1_1|$ and $\langle\tilde{1}_1^{(1)}| \propto \langle 1_1| - \epsilon_1\langle 3_0|$, where vibronic coherences during t_1 are represented by

$$|g_0\rangle\langle\tilde{3}_0^{(1)}| = (1+\epsilon_1^2)^{-1/2}(|g_0\rangle\langle 3_0| + \epsilon_1 |g_0\rangle\langle 1_1|), \tag{43}$$

$$|g_0\rangle\langle\tilde{1}_1^{(1)}| = (1+\epsilon_1^2)^{-1/2}(|g_0\rangle\langle 1_1| - \epsilon_1 |g_0\rangle\langle 3_0|). \tag{44}$$

Here the superindex (1) of $\langle\tilde{3}_0^{(1)}|$ and $\langle\tilde{1}_1^{(1)}|$ reminds us that the vibronic mixing takes place during coherence time t_1: throughout this work, the vibronic eigenstates $\langle\tilde{3}_0^{(2)}|$ and $\langle\tilde{1}_1^{(2)}|$ responsible for the vibronic mixing ϵ_2 during waiting time t_2 have, for the sake of simplicity, been denoted by $\langle\tilde{3}_0|$ and $\langle\tilde{1}_1|$, respectively. We note that ϵ_1 in Eq. (35) is different from ϵ_2 in Eq. (32), as the time evolution of the coherences $|g_0\rangle\langle 3_0|$ and $|g_0\rangle\langle 1_1|$ during coherence time t_1 is governed by a different non-Hermitian operator K_1

$$K_1 = (i\Omega_3 - \Gamma_{g3})|3_0\rangle\langle 3_0| + (i\Omega_1 + i\nu_1 - \Gamma_{g1} - \gamma_\nu)|1_1\rangle\langle 1_1| + i\nu_1\sqrt{S_1}(|3_0\rangle\langle 1_1| + |1_1\rangle\langle 3_0|), \tag{45}$$

defined by $\mathcal{U}(t_1)|g_0\rangle\langle 3_0| = |g_0\rangle\langle 3_0|e^{K_1 t_1}$. In the eigenbasis, the SE diagrams shown in Supplementary Figures 2b-d can be represented by four diagrams shown in Supplementary Figures 5c-f, where the transition dipole moments of $\langle\tilde{3}_0^{(1)}|$ and $\langle\tilde{1}_1^{(1)}|$ are given by $\mu_{3n}(1+\epsilon_1^2)^{-1/2}$ and $-\mu_{3n}(1+\epsilon_1^2)^{-1/2}\epsilon_1$, respectively. It is notable that the vibronic eigenstates $\langle\tilde{3}_0^{(1)}|$ and $\langle\tilde{1}_1^{(1)}|$ during coherence time t_1 are different from the vibronic eigenstates $\langle\tilde{3}_0|$ and $\langle\tilde{1}_1|$ during waiting time t_2, as the vibronic system is in a superposition between electronic ground and excited states (see Eqs. (43) and (44)) and in the electronic excited-state manifold (see Eqs. (41) and (42)), respectively, which leads in general to different values of the vibronic mixings ϵ_1 and ϵ_2. The diagrams shown in Supplementary Figures 5c-f describe the fact that the vibronic eigenstates $\langle\tilde{3}_0^{(1)}|$ and $\langle\tilde{1}_1^{(1)}|$ can be represented by superpositions of $\langle\tilde{3}_0|$ and $\langle\tilde{1}_1|$. In Supplementary Figures 5c and d, for instance, the vibronic eigenstate $\langle\tilde{3}_0^{(1)}|$ induced by the first pulse can be represented by a superposition of $\langle\tilde{3}_0|$ and $\langle\tilde{1}_1|$

$$\langle\tilde{3}_0^{(1)}| = (1+\epsilon_1^2)^{-1/2}(\langle 3_0| + \epsilon_1\langle 1_1|) \tag{46}$$

$$= (1+\epsilon_1^2)^{-1/2}(1+\epsilon_2^2)^{-1/2}[(1+\epsilon_1\epsilon_2)\langle\tilde{3}_0| + (\epsilon_1-\epsilon_2)\langle\tilde{1}_1|)]. \tag{47}$$

Here the prefactors of $\langle\tilde{3}_0|$ and $\langle\tilde{1}_1|$, *i.e.* $(1+\epsilon_1^2)^{-1/2}(1+\epsilon_2^2)^{-1/2}(1+\epsilon_1\epsilon_2)$ and $(1+\epsilon_1^2)^{-1/2}(1+\epsilon_2^2)^{-1/2}(\epsilon_1-\epsilon_2)$, enable us to introduce two separated diagrams shown in Supplementary Figures 5c and d, where the prefactors are multiplied to the response function, similar to the transition dipole moment. Similarly, the other vibronic eigenstate $\langle\tilde{1}_1^{(1)}|$ can be represented by a superposition of $\langle\tilde{3}_0|$ and $\langle\tilde{1}_1|$, leading to the prefactors for the diagrams shown in Supplementary Figures 5e and f. Using the transition dipole moments of $\langle\tilde{3}_0|$ and $\langle\tilde{1}_1|$ induced by the third pulse, one can show that the response function induced by the SE diagrams is given by Eq. (34): here the lineshape functions $(-i(\omega_1-\Omega_3)-\Gamma_{g3})^{-1}$ and $(-i(\omega_1-\Omega_3+\Delta\nu_1)-\Gamma_{g1})^{-1}$ along the ω_1-axis correspond to the diagrams where the vibronic system is in $|g_0\rangle\langle\tilde{3}_0^{(1)}|$ (*cf.* Supplementary Figures 5c and d) and in $|g_0\rangle\langle\tilde{1}_1^{(1)}|$ (*cf.* Supplementary Figures 5e and f), respectively, during coherence time t_1.

The vibronic mixing ϵ_3 during rephasing time t_3 leads to the vibronic eigenstates $|\tilde{3}_0^{(3)}\rangle \propto |3_0\rangle + \epsilon_3|1_1\rangle$ and $|\tilde{1}_1^{(3)}\rangle \propto |1_1\rangle - \epsilon_3|3_0\rangle$, where vibronic coherences during t_3 are represented by

$$|\tilde{3}_0^{(3)}\rangle\langle g_1| = (1+\epsilon_3^2)^{-1/2}(|3_0\rangle\langle g_1| + \epsilon_3|1_1\rangle\langle g_1|), \tag{48}$$

$$|\tilde{1}_1^{(3)}\rangle\langle g_1| = (1+\epsilon_3^2)^{-1/2}(|1_1\rangle\langle g_1| - \epsilon_3|3_0\rangle\langle g_1|). \tag{49}$$

The time evolution of the coherences $|3_0\rangle\langle g_1|$ and $|1_1\rangle\langle g_1|$ is governed by a non-Hermitian operator K_3

$$K_3 = (-i\Omega_3 + i\nu_1 - \Gamma_{g3} - \gamma_v)|3_0\rangle\langle 3_0| + (-i\Omega_1 - \Gamma_{g1})|1_1\rangle\langle 1_1| - i\nu_1\sqrt{S_1}(|3_0\rangle\langle 1_1| + |1_1\rangle\langle 3_0|), \tag{50}$$

defined by $\mathcal{U}(t_3)|3_0\rangle\langle g_1| = e^{K_3 t_3}|3_0\rangle\langle g_1|$. Similar to the SE diagrams, the GSB diagram shown in Supplementary Figure 2e can be represented by four diagrams shown in Supplementary Figures 5g-j. Using the transition dipole moments of the vibronic eigenstates, one can show that the response function induced by the GSB diagrams is given by Eq. (36), where the lineshape functions $(i(\omega_3 - \Omega_1 - \Delta\nu_1) - \Gamma_{g3})^{-1}$ and $(i(\omega_3 - \Omega_1) - \Gamma_{g1})^{-1}$ along the ω_3-axis correspond to the diagrams where the vibronic system is in $|\tilde{3}_0^{(3)}\rangle\langle g_1|$ and in $|\tilde{1}_1^{(3)}\rangle\langle g_1|$, respectively, during t_3.

These results imply that the Feynman diagrams for N11 and R31 can be represented in both uncoupled state basis and vibronic eigenbasis equivalently, and the analytic form of the response functions in Eqs. (31), (34) and (36) is independent of the basis chosen to represent the Feynman diagrams.

2. The response function for N22

Here we provide a vibronic model for bands 2 and 3 of C8O3, where bands 2 and 3 are coupled to the intramolecular vibrational modes with frequency $\hbar\nu_2 \approx 470\,\mathrm{cm}^{-1}$.

In Supplementary Figure 6a, the absolute square of the Fourier transform of the beating signal in N22 is displayed as a function of ω_2, which is normalized by the amplitude of N11 at $\hbar\omega_2 \approx 705\,\mathrm{cm}^{-1}$. The amplitude of N22 is maximized around $\hbar\omega_2 \approx 460\,\mathrm{cm}^{-1}$ with an amplitude in the range of 5 % of the N11 peak. When bands 2 and 3 are coupled to a vibrational mode with frequency ν_2 mediated by an effective Huang-Rhys factor S_2, the response function for N22 is given by

$$R_{1g}(t_2) \approx \mu_{2p}^2 \mu_{3n}^2 \Gamma_{g2}^{-2} \left[e^{(i\Delta\Omega_{32} - \Gamma_{23})t_2} + e^{i\nu_2 t_2} \left(\frac{i\nu_2\sqrt{S_2}}{i\Delta\nu_2 - \Gamma_{23}} \right)^2 \right], \tag{51}$$

with $\Delta\nu_2 = \Delta\Omega_{32} - \nu_2$, μ_{2p} denotes the transition dipole moment of band 2 for light polarized parallel to the longitudinal axis of C8O3 and $\hbar\Gamma_{g2} \approx 110\,\mathrm{cm}^{-1}$ represents the electronic decoherence rate of band 2, both of which can be estimated using the absorption spectrum shown in Supplementary Figure 3. From the experimentally measured beating signal in N22, we found that $\hbar\Gamma_{23} \approx 200\,\mathrm{cm}^{-1} < \hbar(\Gamma_{g2} + \Gamma_{g3})$ (not shown). In Supplementary Figure 6b, the amplitude of the theoretical N22 is displayed as a function of the Huang-Rhys factor S_2, which is about 5 % of N11 over a range of realistic S_2 values. For a comparison, the Huang-Rhys factor S_1 of the vibrational mode with frequency $\hbar\nu_1 \approx 668\,\mathrm{cm}^{-1}$ is marked by a vertical dashed line. These results imply that the small amplitude of the beating signal in N22 is mainly due to the high electronic decoherence rate of band 2.

Supplementary Note 3: Theory of Markovian Correlated Fluctuations

Here we provide a correlated fluctuation model for bands 1 and 3 of C8O3 where coherent interaction between excitons and quasi-resonant vibrations is not considered. Within the level of Markovian quantum master equations, we show that the experimentally measured long-lived beating signals in N11 and R31 cannot be explained by correlated fluctuations.

The main idea of the correlated fluctuations is that when bands 1 and 3 are coupled to a common environment, the correlated noise enables the inter-exciton coherence $|1\rangle\langle 3|$ to decohere very slowly compared to the coherences $|g\rangle\langle 1|$ and $|g\rangle\langle 3|$ between electronic ground state and excitons. This is similar in spirit to the decoherence-free subspaces in quantum information theory[15]. Here we consider a Markovian quantum master equation in the form of

$$\frac{d}{dt}\rho(t) = -\frac{i}{\hbar}[\tilde{H}_e, \rho(t)] + \sum_{\omega}\sum_{\alpha,\beta}\gamma_{\alpha\beta}(\omega)\left(A_\beta(\omega)\rho(t)A_\alpha^\dagger(\omega) - \frac{1}{2}\{A_\alpha^\dagger(\omega)A_\beta(\omega), \rho(t)\}\right), \tag{52}$$

which is the same to Eq. (3.143) in *The Theory of Open Quantum Systems* by H.-P. Breuer and F. Petruccione[12], which is called the Redfield equation with the secular approximation in some literature[16]. Here the interaction Hamiltonian is modeled by $H_{e\text{-ph}} = \sum_\alpha A_\alpha \otimes B_\alpha$ with $A_\alpha = A_\alpha^\dagger$ and $B_\alpha = B_\alpha^\dagger$, each of which is a Hermitian operator of the system and environmental degrees of freedom, respectively. With the exciton states $|k\rangle$ defined by $\tilde{H}_e|k\rangle = \hbar\Omega_k|k\rangle$, we introduce a projection operator $\Pi(\Omega) = \sum_{\Omega=\Omega_k}|k\rangle\langle k| = \sum_k \delta(\Omega, \Omega_k)|k\rangle\langle k|$ where the Kronecker delta is defined by $\delta(i,j) = 1$ if $i = j$ and $\delta(i,j) = 0$ otherwise. In other words, $\Pi(\Omega)$ is a projection operator onto the exciton subspace belonging to the exciton energy Ω. In Eq. (52), $A_\alpha(\omega) = \sum_{\Omega'-\Omega=\omega}\Pi(\Omega)A_\alpha\Pi(\Omega') = \sum_{\Omega,\Omega'}\delta(\omega, \Omega' - \Omega)\Pi(\Omega)A_\alpha\Pi(\Omega')$. The interaction Hamiltonian $H_{e\text{-ph}}$ between excitons and background phonons is modeled by $A_\alpha = |e_\alpha\rangle\langle e_\alpha|$ and $B_\alpha = \sum_\xi \hbar g_{\alpha\xi}(a_\xi^\dagger + a_\xi)$, where $g_{\alpha\xi}$ denotes the coupling of the local excitation of site α to a background phonon mode ξ. When $g_{\alpha\xi} \neq 0$ and $g_{\beta\xi} \neq 0$ for different α and β, spatially separated sites α and β are coupled to a common phonon mode ξ, leading to correlated fluctuations in the energy levels of the different sites α

and β. The correlated fluctuations are absent when each site is coupled to an independent phonon bath, such that $g_{\alpha\xi}g_{\beta\xi} = 0$ for all $\alpha \neq \beta$ and ξ. The information of the correlated fluctuations is included in the definition of $\gamma_{\alpha\beta}(\omega)$ in Eq. (52)

$$\gamma_{\alpha\beta}(\omega) = \frac{1}{\hbar^2}\int_{-\infty}^{\infty} ds e^{i\omega s}\langle B_\alpha^\dagger(s)B_\beta(0)\rangle, \tag{53}$$

where for fixed ω, $\gamma_{\alpha\beta}(\omega)$ form a positive matrix[12]. Here $\gamma_{\alpha\beta}(\omega) = 0$ for all $\alpha \neq \beta$ if each site is coupled to an independent phonon bath and $\gamma_{\alpha\beta}(\omega) \neq 0$ for some $\alpha \neq \beta$ if different sites α and β are coupled to the same phonon modes.

Using experimentally measured absorption and 2D spectra of C8O3, we found that the electronic decoherence rate Γ_{gk} of the coherence $|g\rangle\langle k|$ between electronic ground state and band k is given by $\hbar\Gamma_{g1} \approx 65\,\mathrm{cm}^{-1}$ and $\hbar\Gamma_{g3} \approx 150\,\mathrm{cm}^{-1}$ for bands 1 and 3, respectively. Within the level of the Markovian quantum master equation in Eq. (52), the decoherence rates Γ_{g1} and Γ_{g3} are given by

$$\Gamma_{g1} = \frac{1}{2}\sum_{l\neq 1}\gamma_{1\to l} + \gamma_{g1}, \tag{54}$$

$$\Gamma_{g3} = \frac{1}{2}\sum_{l\neq 3}\gamma_{3\to l} + \gamma_{g3}, \tag{55}$$

where $\gamma_{k\to l}$ denotes the incoherent population transfer rate from band k to band l, and γ_{gk} represents the pure dephasing rate of the coherence $|g\rangle\langle k|$. The population transfer rates $\gamma_{1\to l}$ and $\gamma_{3\to l}$ can be estimated using the exponential dynamics in 2D spectra. To estimate these rates, we performed a global target analysis on all parallel 2D spectra of C8O3[17]. We found that the population transfer rates from band 3 to lower energy bands 1 and 2 are approximately given by $\gamma_{3\to 1} \approx (300\,\mathrm{fs})^{-1}$ and $\gamma_{3\to 2} \approx (66\,\mathrm{fs})^{-1}$, corresponding to $\hbar\gamma_{3\to 1} \approx 18\,\mathrm{cm}^{-1}$ and $\hbar\gamma_{3\to 2} \approx 80\,\mathrm{cm}^{-1}$, respectively, and the other population transfer processes are slow in comparison, *i.e.* $\gamma_{k\to l} \lesssim (2\,\mathrm{ps})^{-1}$. In this case, the pure dephasing rates of $|g\rangle\langle 1|$ and $|g\rangle\langle 3|$ are given by $\hbar\gamma_{g1} \approx \hbar\Gamma_{g1} \approx 65\,\mathrm{cm}^{-1}$ and $\hbar\gamma_{g3} \approx \hbar(\Gamma_{g3} - \frac{1}{2}\gamma_{3\to 1} - \frac{1}{2}\gamma_{3\to 2}) \approx 101\,\mathrm{cm}^{-1}$, respectively.

The electronic decoherence rate Γ_{13} of the inter-exciton coherence $|1\rangle\langle 3|$ between bands 1 and 3 is given by

$$\Gamma_{13} = \frac{1}{2}\sum_{l\neq 1}\gamma_{1\to l} + \frac{1}{2}\sum_{l\neq 3}\gamma_{3\to l} + \gamma_{13}, \tag{56}$$

where γ_{13} is the pure dephasing rate of the inter-exciton coherence in the presence of correlated fluctuations. We found that for given γ_{g1} and γ_{g3}, the inter-exciton dephasing rate γ_{13} should be higher than a theoretical lower bound given by

$$\gamma_{13} \geq \left(\sqrt{\gamma_{g1}} - \sqrt{\gamma_{g3}}\right)^2. \tag{57}$$

Within the level of the Markovian quantum master equation in Eq. (52), the lower bound is not violated by any spectral densities and correlated fluctuations, as will be shown below. Using the estimated values of the pure dephasing rates $\hbar\gamma_{g1} \approx 65\,\mathrm{cm}^{-1}$ and $\hbar\gamma_{g3} \approx 101\,\mathrm{cm}^{-1}$, we found that the lower bound in Eq. (57) is reduced to $\hbar\gamma_{13} \gtrsim 4\,\mathrm{cm}^{-1}$. Therefore, even in the presence of correlated fluctuations, the decoherence rate Γ_{13} of the inter-exciton coherence $|1\rangle\langle 3|$ in Eq. (56) should be higher than a lower bound given by

$$\Gamma_{13} \geq \frac{1}{2}(\gamma_{3\to 1} + \gamma_{3\to 2}) + \left(\sqrt{\gamma_{g1}} - \sqrt{\gamma_{g3}}\right)^2 \approx (100\,\mathrm{fs})^{-1}. \tag{58}$$

It is notable that the lowest decoherence rate $\Gamma_{13} \approx (100\,\mathrm{fs})^{-1}$ (*cf.* $\hbar\Gamma_{13} \approx 53\,\mathrm{cm}^{-1}$) is too high to explain the long-lived beating signals observed in the experiment, as shown in Supplementary Figure 7a, where the simulated results based on the correlated fluctuation model are shown as a blue solid line and the experimental results are shown as a light blue line. These results are mainly due to the fast population transfer from band 3 to bands 1 and 2 observed in the experiment.

We note that our results are not sensitive to the estimated values of the population transfer rates. The inter-exciton decoherence rate Γ_{13} is minimized when there is no population transfer between excitons, *i.e.* $\gamma_{k\to l} = 0$ for all $k \neq l$, where the coherences between electronic ground state and excitons are destroyed only by pure dephasing noise, *i.e.* $\hbar\Gamma_{g1} = \hbar\gamma_{g1} \approx 65\,\mathrm{cm}^{-1}$ and $\hbar\Gamma_{g3} = \hbar\gamma_{g3} \approx 150\,\mathrm{cm}^{-1}$. Even though this condition is not satisfied for C8O3, this is the best scenario of the correlated fluctuation model where the decoherence rate Γ_{13} of the inter-exciton coherence is minimized

$$\Gamma_{13} = \gamma_{13} \geq \left(\sqrt{\gamma_{g1}} - \sqrt{\gamma_{g3}}\right)^2 \approx (303\,\mathrm{fs})^{-1}. \tag{59}$$

However, even in this case, the lowest decoherence rate $\Gamma_{13} \approx (303\,\mathrm{fs})^{-1}$ supported by correlated fluctuations is not low enough to explain the experimentally measured long-lived beating signals in N11 and R31, which persist beyond $t_2 \approx 800\,\mathrm{fs}$, as shown in

Supplementary Figure 7b. This is due to the different decoherence rates $\Gamma_{g1} < \Gamma_{g3}$ of $|g\rangle\langle 1|$ and $|g\rangle\langle 3|$ observed in the experiment. This leads to a non-zero lower bound on the inter-exciton decoherence rate Γ_{13}, as shown in Eq. (59). In addition, the beating signals in N11 and R31 consist of a short-lived component with 1/e decay time of ~ 66 fs as well as a long-lived component persisting up to $t_2 \approx 1$ ps. This is contrary to the prediction of the correlated fluctuation model where a single oscillatory component is expected with 1/e decay time of Γ_{13}^{-1}. For a comparison, the theoretical prediction of the vibronic model is shown in Supplementary Figure 7c, where both short-lived and long-lived components are present. We note that in the vibronic model, the decay rate of the long-lived component is independent of Γ_{g1} and Γ_{g3}, as it is determined by the other degrees of freedom, such as the dissipation rate γ_v of the vibrations and the degree of vibronic mixing ϵ_2 leading to a lifetime borrowing effect $\delta\gamma$, as shown in Eq. (30).

We now derive Eqs. (54)-(57) using the Markovian quantum master equation in Eq. (52).

(Dephasing noise) We start with the case that $\omega = 0$, leading to the pure dephasing noise. For the sake of simplicity, we assume that there is no degeneracy in the exciton energies Ω_k, such that $\Omega_k \neq \Omega_l$ for all $k \neq l$. In this case, $A_\alpha(0) = \sum_k |k\rangle\langle k| A_\alpha |k\rangle\langle k| = \sum_k |\langle k|e_\alpha\rangle|^2 |k\rangle\langle k|$. By substituting $|g\rangle\langle 1|$, $|g\rangle\langle 3|$ and $|1\rangle\langle 3|$ to the dissipator of the quantum master equation for $\omega = 0$

$$\frac{d}{dt}\rho(t) = \sum_{\alpha,\beta} \gamma_{\alpha\beta}(0) \left(A_\beta(0)\rho(t)A_\alpha^\dagger(0) - \frac{1}{2}\{A_\alpha^\dagger(0)A_\beta(0), \rho(t)\} \right), \tag{60}$$

one obtains the following pure dephasing rates of the coherences $|g\rangle\langle 1|$, $|g\rangle\langle 3|$ and $|1\rangle\langle 3|$

$$\gamma_{g1} = \frac{1}{2}\sum_{\alpha,\beta} |\langle 1|e_\alpha\rangle|^2 \gamma_{\alpha\beta}(0) |\langle 1|e_\beta\rangle|^2 = |\vec{v}_1|^2, \tag{61}$$

$$\gamma_{g3} = \frac{1}{2}\sum_{\alpha,\beta} |\langle 3|e_\alpha\rangle|^2 \gamma_{\alpha\beta}(0) |\langle 3|e_\beta\rangle|^2 = |\vec{v}_3|^2, \tag{62}$$

$$\gamma_{13} = \frac{1}{2}\sum_{\alpha,\beta} \left(|\langle 1|e_\alpha\rangle|^2 - |\langle 3|e_\alpha\rangle|^2\right) \gamma_{\alpha\beta}(0) \left(|\langle 1|e_\beta\rangle|^2 - |\langle 3|e_\beta\rangle|^2\right) = |\vec{v}_1 - \vec{v}_3|^2, \tag{63}$$

where $\vec{v}_k$ represents a vector defined by $\vec{v}_k = 2^{-1/2}\hat{\gamma}^{1/2}\vec{w}_k$: here $\vec{w}_k$ is a real vector with elements $|\langle k|e_\alpha\rangle|^2 \geq 0$ and $\hat{\gamma}$ is a positive matrix[12] with elements $\gamma_{\alpha\beta}(0)$, leading to to a positive matrix $\hat{\gamma}^{1/2}$ defined by $\hat{\gamma} = \hat{\gamma}^{1/2}\hat{\gamma}^{1/2}$. For given pure dephasing rates $\gamma_{g1} = |\vec{v}_1|^2$ and $\gamma_{g3} = |\vec{v}_3|^2$, the inter-exciton pure dephasing rate γ_{13} is constrained by

$$\gamma_{13} = |\vec{v}_1 - \vec{v}_3|^2 \geq (|\vec{v}_1| - |\vec{v}_3|)^2 = \left(\sqrt{\gamma_{g1}} - \sqrt{\gamma_{g3}}\right)^2, \tag{64}$$

due to the triangle inequality, $|\vec{v}_1 - \vec{v}_3| + |\vec{v}_3| \geq |\vec{v}_1|$ and $|\vec{v}_1 - \vec{v}_3| + |\vec{v}_1| \geq |\vec{v}_3|$. Here the equality holds if and only if $\vec{v}_1$ is parallel to $\vec{v}_3$, which depends on $\hat{\gamma}^{1/2}$ (spectral densities and correlated fluctuations) as well as $\vec{w}_1$ and $\vec{w}_3$ (the spatial overlap between excitonic wavefunctions). Note that the lower bound in Eq. (64) has been derived based on the positivity of $\hat{\gamma}^{1/2}$, which is satisfied for any spectral densities and correlated fluctuations. These results imply that the inter-exciton dephasing rate γ_{13} can be reduced by the correlated fluctuations as well as the spatial overlap between excitonic wavefunctions.

(Exciton relaxation) We now consider the case that $\omega \neq 0$, leading to the incoherent population transfer between excitons. With $\Delta\Omega_{kl} = \Omega_k - \Omega_l$ denoting the exciton energy splitting between bands k and l, $A_\alpha(\omega) = \sum_{k,l} \delta(\omega, \Delta\Omega_{kl}) |l\rangle\langle l| A_\alpha |k\rangle\langle k| = \sum_{k,l} \delta(\omega, \Delta\Omega_{kl}) \langle l|e_\alpha\rangle\langle e_\alpha|k\rangle |l\rangle\langle k|$ and the dissipator of the quantum master equation for $\omega \neq 0$ is given by

$$\frac{d}{dt}\rho(t) = \sum_{k\neq l}\sum_{\alpha,\beta} \gamma_{\alpha\beta}(\Delta\Omega_{kl}) \left(A_\beta(\Delta\Omega_{kl})\rho(t)A_\alpha^\dagger(\Delta\Omega_{kl}) - \frac{1}{2}\{A_\alpha^\dagger(\Delta\Omega_{kl})A_\beta(\Delta\Omega_{kl}), \rho(t)\} \right), \tag{65}$$

where the population transfer rate $\gamma_{k\to l}$ from band k to band l is given by

$$\gamma_{k\to l} = \sum_{\alpha,\beta} \langle k|e_\alpha\rangle\langle e_\alpha|l\rangle \gamma_{\alpha\beta}(\Delta\Omega_{kl}) \langle l|e_\beta\rangle\langle e_\beta|k\rangle \geq 0, \tag{66}$$

which is positive, as $\gamma_{\alpha\beta}(\Delta\Omega_{kl})$ form a positive matrix[12] for given $\Delta\Omega_{kl}$. By substituting $|g\rangle\langle 1|$, $|g\rangle\langle 3|$ and $|1\rangle\langle 3|$ to the dissipators in Eqs. (60) and (65), one can show that the electronic decoherence rates Γ_{g1}, Γ_{g3} and Γ_{13} satisfy Eqs. (54)-(57). These results are valid for any spectral densities and correlated fluctuations within the level of the Markovian quantum master equation in Eq. (52), which includes the theoretical models considered in previous studies[16].

In summary, the correlated fluctuation model cannot explain the long-lived beating signals in N11 and R31 within the level of Markovian quantum master equations, as the decoherence rate Γ_{13} of the inter-exciton coherence $|1\rangle\langle 3|$ is constrained by the experimentally observed asymmetric decoherence rates Γ_{g1} and Γ_{g3}, *i.e.* $\Gamma_{g3} \approx 2\Gamma_{g1}$, and the fast population transfer from band 3 to bands 1 and 2. We note that the asymmetric decoherence rates Γ_{g1} and Γ_{g3} are related to the fact that i) the lineshape of R31 is elongated along ω_1-axis (*cf.* Figures 2b and d in the main text), ii) the amplitudes of the short-lived beating signals in N11 and R31 are different in magnitude (*cf.* Figures 3b and c in the main text), and iii) the absorptive linewidths of bands 1 and 3 are different (*cf.* Supplementary Figure 3).

Supplementary References:

[1] Dékány, Gy., Csóka, I. & Erös, I. Adsorption of neutral polymers on negatively charged liposomes. A novel quantitative method to measure the rate of polymer adsorption on the liposomal surface. *Colloid Polym. Sci.* **279**, 966–975 (2001).

[2] Eisele, D. M. *et al*. Robust excitons inhabit soft supramolecular nanotubes. *PNAS* **111**, E3367–E3375 (2014).

[3] Von Berlepsch, H. *et al*. Stabilization of individual tubular J-aggregates by poly(vinyl alcohol). *J. Phys. Chem. B* **107**, 14176–14184 (2003).

[4] Tauber, M. J., Mathies, R. A., Chen, X. & Bradforth, S. E. Flowing liquid sample jet for resonance Raman and ultrafast optical spectroscopy. *Rev. Sci. Instrum.* **74**, 4958–4960 (2003).

[5] Brixner, T., Mančal, T., Stiopkin, I. V. & Fleming, G. R. Phase-stabilized two-dimensional electronic spectroscopy. *J. Chem. Phys.* **121**, 4221–4236 (2004).

[6] Augulis, R. & Zigmantas, D. Two-dimensional electronic spectroscopy with double modulation lock-in detection: enhancement of sensitivity and noise resistance. *Opt. Express* **19**, 13126–13133 (2011).

[7] Hochstrasser, R. M. Two-dimensional IR-spectroscopy: polarization anisotropy effects. *Chem. Phys.* **266**, 273–284 (2001).

[8] Schlau-Cohen, G. S. *et al*. Elucidation of the timescales and origins of quantum electronic coherence in LHCII. *Nature Chem.* **4**, 389–395 (2012).

[9] Westenhoff, S. Paleček, D., Edlund, P., Smith, P. & Zigmantas, D. Coherent picosecond exciton dynamics in a photosynthetic reaction center. *J. Am. Chem. Soc.* **134**, 16484–16487 (2012).

[10] Jonas, D. M. Two-dimensional femtosecond spectroscopy. *Annu. Rev. Phys. Chem.* **54**, 425–463 (2003).

[11] Milota, F., Lincoln, C. N. & Hauer, J. Precise phasing of 2D-electronic spectra in a fully non-collinear phase-matching geometry. *Opt. Express* **21**, 15904–15911 (2013).

[12] Breuer, H. & Petruccione, F. *The theory of open quantum systems* (Oxford University Press, 2002).

[13] Didraga, C. *et al*. Structure, spectroscopy, and microscopic model of tubular carbocyanine dye aggregates. *J. Phys. Chem. B* **108**, 14976–14985 (2004).

[14] Tiwari, V., Peters, W. K. & Jonas, D. M. Electronic resonance with anticorrelated pigment vibrations drives photosynthetic energy transfer outside the adiabatic framework. *PNAS* **110**, 1203–1208 (2013).

[15] Lidar, D. A., Chuang, I. L. & Whaley, K. B. Decoherence-free subspaces for quantum computation. *Phys. Rev. Lett.* **81**, 2594 (1998).

[16] Rebentrost, P., Mohseni, M. & Aspuru-Guzik, A. Role of quantum coherence and environmental fluctuations in chromophoric energy transport. *J. Phys. Chem. B* **113**, 9942–9947 (2009).

[17] Milota, F. *et al*. Vibronic and vibrational coherences in two-dimensional electronic spectra of supramolecular J-aggregates. *J. Phys. Chem. A* **117**, 6007–6014 (2013).
